# Proteomic Analysis of Human Serum for Patients at Different Pathological Stages of Hepatic Fibrosis

**DOI:** 10.1155/2021/3580090

**Published:** 2021-11-28

**Authors:** Kang Zhao, Jucun Huang, Hongmei Xia, Jianjun Zhang, Liming Liu

**Affiliations:** Department of Hepatology, Hubei No.3 People's Hospital of Jianghan University, Wuhan 430033, China

## Abstract

**Background:**

Hepatic fibrosis is a severe liver disease that has threatened human health for a long time. In order to undergo timely and adequate therapy, it is important for patients to obtain an accurate diagnosis of fibrosis. Laboratory inspection methods have been efficient in distinguishing between advanced hepatic fibrosis stages (F3, F4), but the identification of early stages of fibrosis has not been achieved. The development of proteomics may provide us with a new direction to identify the stages of fibrosis.

**Methods:**

We established serum proteomic maps for patients with hepatic fibrosis at different stages and identified differential expression of proteins between fibrosis stages through ultra-high-performance liquid chromatography tandem mass spectrometry proteomic analysis.

**Results:**

From the proteomic profiles of the serum of patients with different stages of liver fibrosis, a total of 1,338 proteins were identified. Among three early fibrosis stages (control, F1, and F2), 55 differential proteins were identified, but no proteins simultaneously exhibited differential expression between control, F1, and F2. Differential proteins were detected in the comparison between different fibrosis stages. Significant differences were found between advanced fibrosis stages (F2-vs.-F3 and F3-vs.-F4) through a series of statistical analysis, including hierarchical clustering, Gene Ontology (GO) functional annotation, Kyoto Encyclopedia of Genes and Genomes pathway, and protein-protein interaction network analysis. The differential proteins identified by GO annotation were associated with biological processes (mainly platelet degranulation and cell adhesion), molecular functions, and cellular components.

**Conclusions:**

All potential biomarkers identified between the stages of fibrosis could be key points in determining the fibrosis staging. The differences between early stages may provide a useful reference in addressing the challenge of early fibrosis staging.

## 1. Introduction

Hepatic fibrosis is caused by chronic damage to the liver and is often accompanied by the excessive accumulation of extracellular matrix proteins including collagen, which occurs in most types of chronic liver diseases [1]. In the pathological process, the normal physiological function of the liver gradually degenerates, and the subsequent formation of liver pseudolobules results in cirrhosis [2]. Historically, the development of hepatic fibrosis has been considered as an irreversible process because of the alteration in hepatic parenchymal structures [3, 4]. However, Soyer et al. reported that advanced liver fibrosis is potentially reversible [5]. Hepatic stellate cells, which are a type of liver stromal cells, were first identified as the major collagen-producing cells in the liver in the 1980s, and their activation was considered the key link to hepatic fibrosis formation [6, 7].

Several studies have utilized parameters such as backscatter coefficient, speed of sound, attenuation coefficient, spectral slope, and mean scatterer spacing to characterize hepatic tissues [8–13]. These parameters could be efficient in differentiating between normal and pathological organs. In recent decades, investigators have focused more attention in the exploration of pathological changes between the different pathological stages of hepatic fibrosis, of which five have been identified. Even though diagnostic approaches for hepatic fibrosis have achieved great progress, liver biopsy still acts as the “golden rule” to assess the pathological stage of liver fibrosis [14]. However, possible errors exist in the evaluation of fibrosis stage through liver biopsy [15]. As a result, this technique cannot be applied as a conventional method in medical intervention. Other detection methods complementary to liver biopsy have been recommended to quantify fibrosis, such as fibrotest and elastographic methods. It is critical for fibrosis patients to obtain an accurate diagnosis in the early stages to receive adequate therapy. However, an overlap between early stages of fibrosis (control/F0, F1, and sometimes F2) has been discovered by both elastographic methods and ultrasonic approaches [10, 11, 16–18]. It is difficult to differentiate between the F0 and F1 (and sometimes F2) stages, but the identification of changes between early stages is essential for doctors to determine proper treatment schemes for fibrosis patients. To address this challenge, it is necessary to establish a convenient and precise differential method to diagnose the different stages of hepatic fibrosis.

Proteomics, a new “omics” approach proposed by Wilkins and Williams in 1994, offers a method of exploring whole gene products in various organs [19], showing great advantages of genomic sequencing and protein mapping [20]. In recent years, proteomics has been widely applied in many areas of study, especially in pharmaceuticals and cancer research. Fedchenko et al. researched the proteomic profiling data of HEK293 protein and found that the C-terminal region is vital for specific binding of renalase between its target and receptors [21]. Cao et al. used proteomic technologies to gain a better understanding of multidrug resistance mechanisms in cancer [22]. Anuli and Aebersold applied targeted proteomic analysis to advance translational research and precision medicine, providing a new way to develop precise therapeutics for future clinical applications [23]. Kosteria et al. utilized mass spectrometry-based proteomics to research pediatric endocrine and metabolic diseases for the prevention of disease manifestation or future complications [24].

For investigating liver disease more comprehensively, proteomic approaches have been utilized to study liver cirrhosis and fibrosis. Based on proteomic analysis, Xu et al. found that dioscin, a potent inhibitor of integrin *α*5, reduced collagen synthesis and exerted protective effects against liver fibrosis [25]. Ahmad and Ahmad analyzed the serum and liver proteome profiles in rats subjected to N′-nitrosodimethylamine-induced hepatic fibrosis and treated with resveratrol, demonstrating that the oxidative pathway was the main mediator in both experimental hepatic fibrosis [26]. Bhagwat et al. performed a quantitative targeted proteomics study to explore the abundance of drug-metabolizing enzymes in cirrhotic livers. The study illustrated that protein abundance data combined with physiologically based pharmacokinetic modeling acted as a drug disposition predicter in special populations [27]. Prasad et al. carried out proteomic-genomic analysis to elucidate the pathways and networks involved in liver fibrosis, with the aim of solving key clinical questions [28]. Golizeh et al. performed proteomic fingerprinting analysis to investigate serum proteome profiles at different stages of fibrosis in patients coinfected with human immunodeficiency virus/hepatitis C virus (HIV/HCV) or mono-infected with HCV. Proteomic profiling was efficiently applied to identify diagnostic serum biomarkers of fibrosis (F1 vs. F3/4) in both HIV/HCV coinfected and HCV mono-infected individuals [29]. Huang et al. conducted serum proteomic analysis between hepatitis B virus-infected patients and carriers. They screened out 13 differential biomarkers between the two groups and pointed out that а-enolase and thrombospondin could be serum biomarkers in the clinical diagnosis of hepatic fibrosis [30]. Although researchers have investigated the different stages of hepatic fibrosis by proteomic analysis, they mainly focused on identifying and diagnosing the F1 vs. F3/4 stage. Few proteomic research studies have been performed to explore the difference among all five stages (F0-F4) and the three early stages (F0-F3) of hepatic fibrosis, even though the accurate identification of fibrosis is important for determining proper treatment.

In the present study, ultra-high-performance liquid chromatography tandem mass spectrometer (UPLC-MS/MS) was utilized to explore the comprehensive proteomic profiles of the serum in patients at five different stages (F0-F4) of hepatic fibrosis. This study is aimed at screening for protein biomarkers of different stages, especially the three early stages of hepatic fibrosis.

## 2. Materials and Methods

### 2.1. Serum Sample Collection

Blood samples were collected from two healthy volunteers and eight patients at four pathological stages of hepatic fibrosis (two each), labeled as F1, F2, F3, and F4. Blood samples from healthy volunteers served as controls. The experimental procedures were conducted under laboratory conditions.

The collected blood was centrifuged at 4000 rpm for 5 min at 4°C immediately upon extraction, and approximately 1 mL of the supernatant was pipetted into another tube for further study. Each sample was clearly labeled.

The stages of fibrosis were classified by an experienced pathologist according to the Meta-analysis of Histological Data in Viral Hepatitis (METAVIR criteria): control for the absence of fibrosis, F1 for portal fibrosis without septa, F2 for portal fibrosis with few septa, F3 for septal fibrosis without cirrhosis, and F4 for cirrhosis [31].

### 2.2. Sample Preparation

Twelve high-abundance proteins from 10 *μ*L human serum were removed using Thermo Scientific Pierce Top 12 Abundant Protein Depletion Spin Column according to the protocol. The protein solution was concentrated by ultrafiltration tube with a molecular weight cut-off of 10 kDa. The protein concentration in the supernatant was determined using a bicinchoninic acid assay, and 50 *μ*g of protein per condition was transferred into a new filter and adjusted to a final volume of 100 *μ*L with 100 mM triethylammonium bicarbonate. The sample was then incubated with 5 *μ*L of 200 mM dithiothreitol at 37°C for 2 h, and 5 *μ*L of 575 mM iodoacetamide was added. The sample was further incubated for 1 h in the absence of light at room temperature.

### 2.3. Protein Digestion and Tandem Mass Tag (TMT) Labeling

Proteins were digested with serial grade modified trypsin (Promega, Madison, WI), and the resulting peptide mixture was labeled with the TMT10 kit. The mixed labeled samples were then desalted using a C18 solid phase extraction column (Sep-Pak C18, Waters Corporation, Milford, MA) and dried in a vacuum desiccator.

### 2.4. High-pH Reverse-Phase Separation

Experimental methods were based on the following reference [32]. The mixed sample was redissolved in buffer A (10 mM ammonium formate in water, pH 10.0, adjusted with ammonium hydroxide) and performed using a linear gradient from 0% to 45% of B (10 mM ammonium formate in 90% acetonitrile, pH 10.0, adjusted with ammonium hydroxide) in 35 min. The Aquity UPLC system (Waters Corporation) connected to a reverse-phase column (BEH C18 column, 2.1 mm × 150 mm, 1.7 *μ*m, 300 Å, Waters Corporation) was used for fractionation. The flow rate was maintained at 250 *μ*L/min, and the temperature was maintained at 45°C. Twelve fractions were collected and dried in a vacuum concentrator for the next step.

### 2.5. Low-pH Nano-UPLC-MS/MS Analysis

The fractions were resuspended in 35 *μ*L of 0.1% formic acid, separated by nanoLC, and analyzed by online nanoelectrospray tandem mass spectrometry (MS) (Thermo Fisher Scientific, San Jose, CA). 5 *μ*L of samples was loaded onto the trap column (Thermo Scientific Acclaim PepMap C18, 100 *μ*m × 2 cm) with a flow of 10 *μ*L/min for 3 min and subsequently separated on an analytical column (Acclaim PepMap C18, 75 *μ*m × 25 cm) with a linear gradient from 2% to 30% D (ACN with 0.1% formic acid) in 80 min. The flow rate was maintained at 300 nL/min, and an electrospray voltage of 2.0 kV versus the inlet of the MS was used.

The Orbitrap Fusion MS was operated in data-dependent mode with automatic switching between MS and MS/MS acquisition, the detail experimental procedures were performed following the reference [33].

### 2.6. Database Search

Tandem MS were extracted using the Proteome Discoverer software (version 1.4.0.288, Thermo Fisher Scientific). Charge state deconvolution and de-isotropy were not performed. All MS/MS samples were analyzed with Mascot (version 2.3, Matrix Science, London, UK). Mascot was set to search the Uniprot-SwissProt database (Taxonomy: Homo sapiens, 20259 entries) assuming trypsin as the digestive enzyme. Mascot was searched with a fragment ion mass tolerance of 0.050 Da and a parent ion tolerance of 10.0 PPM. Carbamidomethyl of cysteine and TMT 6plex of lysine and the n-terminus were specified in Mascot as fixed modifications. Oxidation of methionine was specified in Mascot as a variable modification.

### 2.7. Quantitative Data Analysis

A percolator algorithm was used to control the false discovery rates of peptides at lower than 1%. The TMT 10plex quantification method was used to calculate the reported quantification ratios from the values of the different quantification channels. Only unique peptides were used for protein quantification, and the method of normalization to the protein median was used to correct experimental bias; the minimum number of proteins that must be observed was set to 200. *P* < 0.1 and fold change < 0.83 or >1.2 were chosen to screen out differential protein biomarkers.

Hierarchical clustering was performed by the Heatmap illustrator tool. Functional annotation with Gene Ontology (GO) analysis was conducted in the GO database (http://geneontology.org), and DAVID 6.8 (http://david.abcc.ncifcrf.gov/) was applied for functional enrichment analysis. Analysis of metabolic pathways of differential proteins was carried out using the Kyoto Encyclopedia of Genes and Genomes (KEGG) (http://www.kegg.jp/kegg/pathway.html). The protein-protein interaction (PPI) network was constructed in the STRING database (version 10, http://string-db.org).

## 3. Results

### 3.1. Proteome of Hepatic Fibrosis

In the present study, two biological replicates were included for each pathologic stage of hepatic fibrosis in the proteomic analysis to improve the reliability of the experimental data. Comprehensive statistical analysis was performed to screen out different proteins. A total of 331,767 spectra were generated. The number of matched spectra was 26,063, with 6,688 peptides and 1,338 proteins belonging to 762 protein groups. Among these biomarkers, we detected 17 differential proteins between F1 and control, where 8 showed downregulation in the control group and the other 9 showed much higher expression in the control group. For the comparison between F1 and F2, we screened out 23 differential biomarkers, where 7 revealed a decreasing trend in F1 fibrosis stage, and 16 showed an increase. A total of 21 proteins were selected as differential biomarkers between F2 and control, among which 10 revealed a decrease in F2 and 11 showed the opposite trend compared to the control group. Detailed information on the differential proteins among the three early hepatic fibrosis stages is presented in Table 1. Among the 55 detected proteins, two (P6, P8) showed differences in both F1 and F2 compared to those in the control group. P10 and P11 revealed an increase in F1 compared to those in F2. P23 and P37 showed differential expression in F2 compared to those in control and F1. However, no proteins were found to simultaneously exhibit differential expression between control, F1, and F2.

Otherwise, a total of 41 proteins were identified as potential biomarkers between F2 and F3. The expression abundance of 23 proteins in serum was decreased in the F2 stage, whereas 18 other proteins showed an increasing trend (Table 2). Between F3 and F4, 24 proteins showed lower abundance in F3, and 22 were increased in F3 compared to F4 (Table 3). The descriptions of the differential proteins are listed in the Supplementary file (Table S1).

### 3.2. Hierarchical Clustering Analysis of Differential Proteins

**Figure 1:**
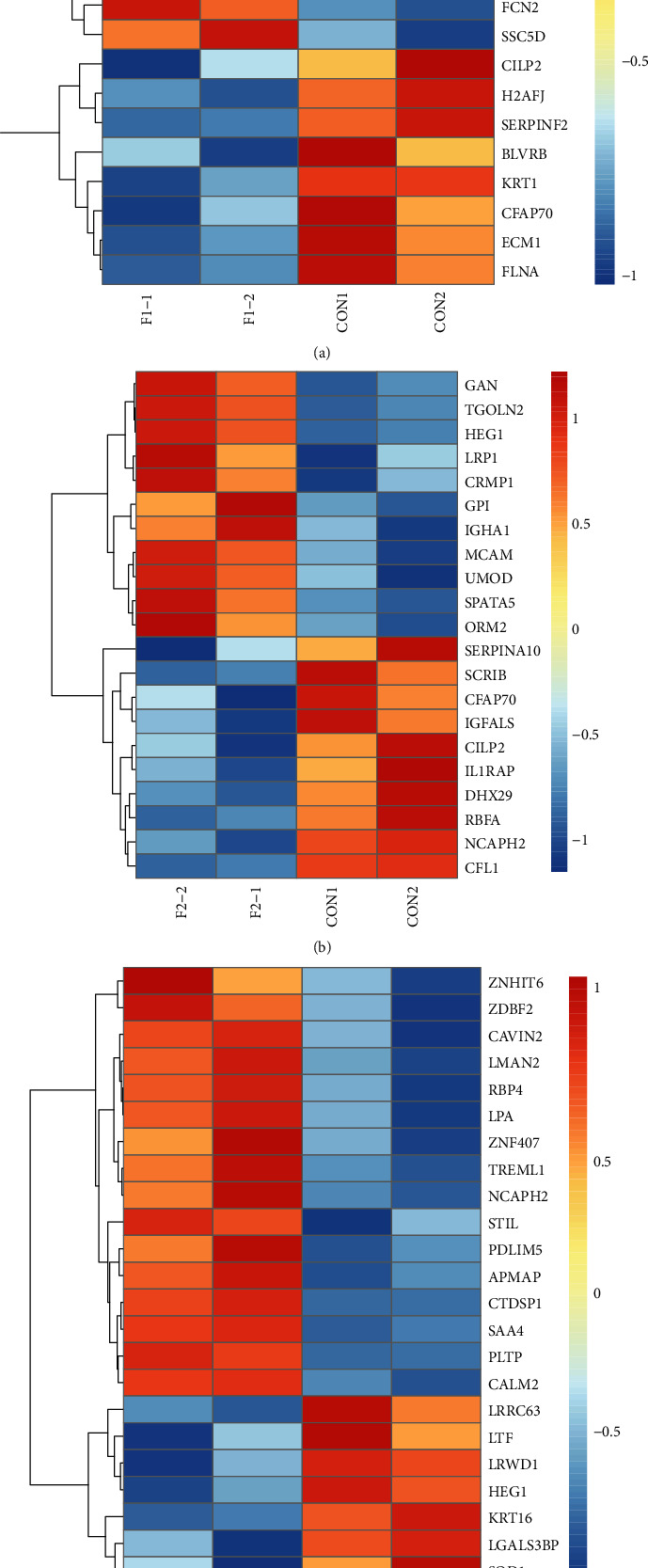
Heat maps of differential proteins between two early stages of hepatic fibrosis: (a) F1 compared to CON; (b) F2 compared to CON; (c) F1 compared to F2. Each line represents a differential protein, and each cross represents a serum sample group. Colors indicate the abundance intensity, with higher abundance intensity represented by a gradual increase from blue to red.

To obtain a visual illustration of differential serum proteins between the hepatic fibrosis stages, a hierarchical clustering analysis was conducted to display the differences between the stages. From the heat maps, we distinguished clear differences in protein expression abundance between F1 and control, as well as in other group comparisons (Figure 1).

### 3.3. Functional Annotation of Proteins with GO

**Figure 2:**
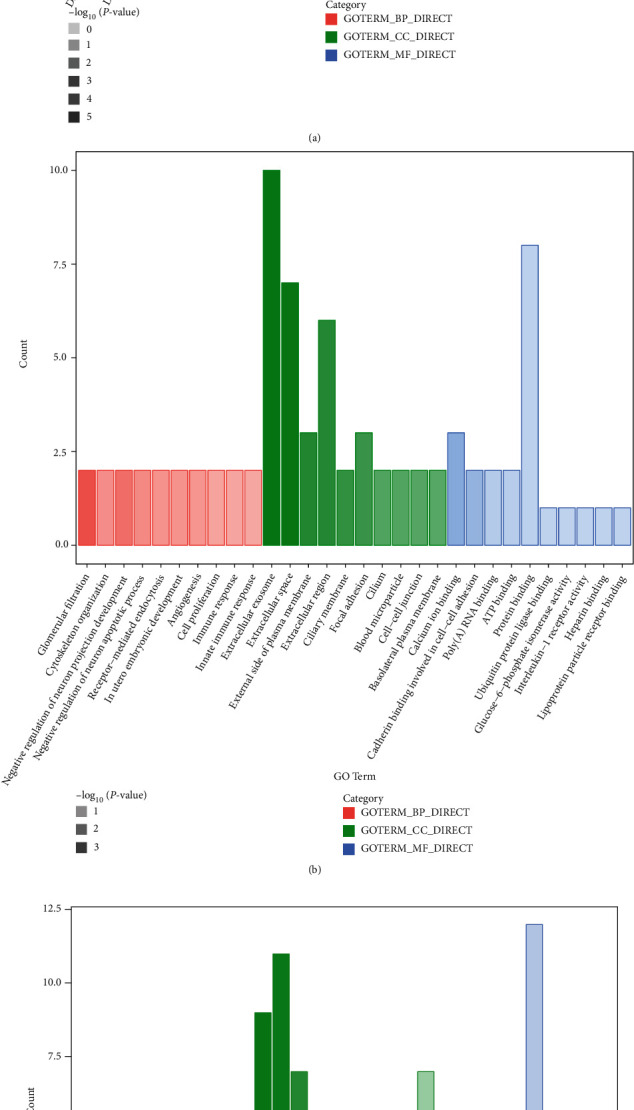
Functional annotation of proteins with GO between two early stages of hepatic fibrosis: (a) F1 compared to CON; (b) F2 compared to CON; (c) F1 compared to F2. Pink, green, and blue bars represent proteins functionally annotated for biological processes, cellular components, and molecular functions, respectively.

To comprehensively summarize functional gene information, GO was conducted to reveal the functional category of genes associated with differential expression. The analytical results are partly presented on the top ten functional categories. Comparing F1 with the control, 172 GO terms were annotated in biological processes, which mainly consisted of platelet degranulation, fibrinolysis, and acute-phase response. 70 GO terms were identified in cellular components, with the highest number of proteins associated with the extracellular exosomes, extracellular space, and plasma membrane. The number of molecular functions represented by proteins was 71, with the highest number of proteins associated with protein binding. Detail information is presented in Figure 2(a) and Supplementary Table S2. Between F2 and control, 159 GO terms were annotated in biological processes (cytoskeleton organization, immune response, and innate immune response), 68 were identified in molecular functions (extracellular exosomes, extracellular space, and extracellular region), and 41 were identified in molecular functions (protein binding and calcium ion binding) (Figure 2(b) and Supplementary Table S3). In comparing between F1 and F2, the number of proteins associated with biological processes, cellular components, and molecular functions was 239, 67, and 64, respectively (Figure 2(c) and Supplementary Table S4). Furthermore, information on the GO functional annotation between F2 and F3 and between F3 and F4 is presented in the Supplementary files (Figure S1 and S2, Tables S5 and S6).

### 3.4. KEGG Pathway Analysis of the Identified Differential Proteins

The metabolic pathway analysis of differentially expressed proteins reveals significant systematic changes in signaling pathways under different experimental processes. Based on the results of KEGG analysis, no differential metabolic pathway was found between F1 and control and between F2 and control. Cholesterol metabolism was the unique differential pathway identified between F1 and F2. Otherwise, in the comparison between F2 and F3, 14 metabolic pathways were detected, including extracellular matrix-receptor interaction, protein digestion and absorption, and microRNAs in cancer. Between F3 and F4, we identified 19 differential metabolic pathways, including pathways in cancer and leukocyte transendothelial migration. Detailed information is presented in the Supplementary files (Table S7).

### 3.5. PPI Network Analysis of Identified Differential Proteins

**Figure 3:**
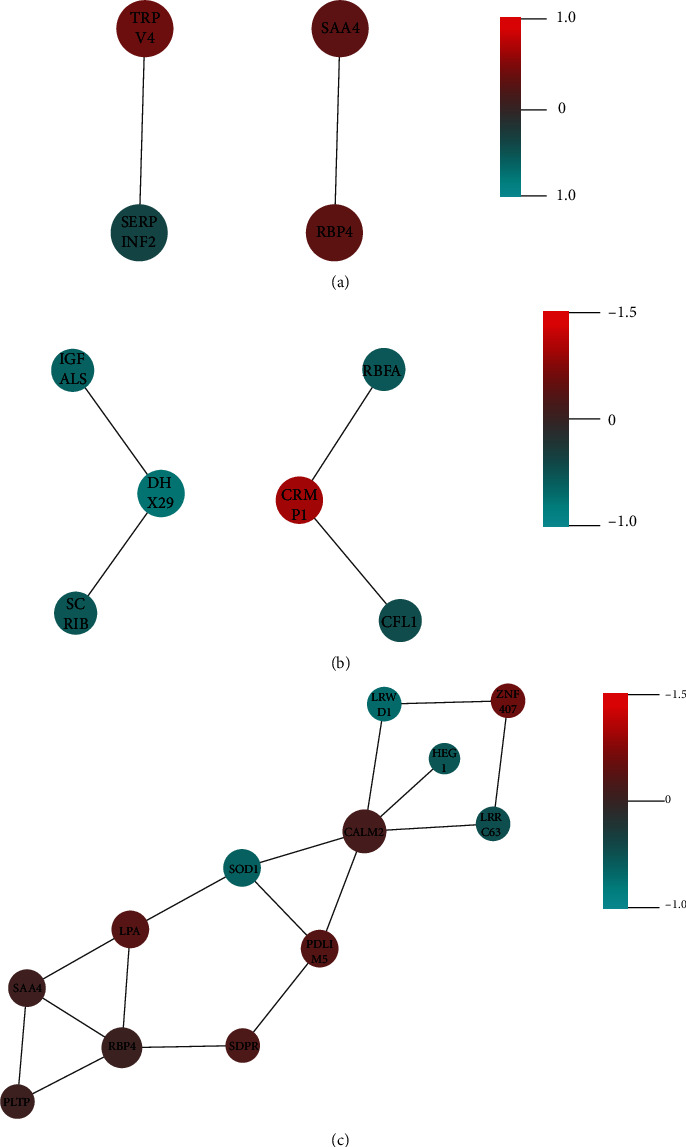
PPI of differentially expressed proteins between two early stages of hepatic fibrosis: (a) F1 compared to CON; (b) F2 compared to CON; (c) F1 compared to F2. Colors indicate the abundance intensity, with higher abundance intensity represented by a gradual increase from blue to red. The size of the circles represents the degree of differentially expressed proteins, with larger circles indicating higher degrees.

Proteins that function in biological processes can be analyzed to construct a PPI network to explore the changes in differentially expressed proteins at the proteome level. Comparing between F1 and control, four proteins were involved in PPI relationships, all with a single degree. Between F2 and control, four proteins with one degree and two with two degrees were detected in the PPI network. Twelve proteins were identified in the PPI network analysis between F1 and F2, among which the degree number ranged from 1 to 5. Detailed information on the PPI network analysis between hepatic fibrosis stages is shown in Figure 3 and the Supplementary files (Table S8). The results of PPI network analysis between F2 and F3 and between F3 and F4 are also presented in the Supplementary files (Table S8, Figure S3 and S4).

## 4. Discussion

Hepatic cirrhosis patients undergo a series of pathological processes through the various stages of hepatic fibrosis, but the specific mechanisms of action are still unclear. Fontana et al. analyzed the current developments in the detection of differential serum biomarkers and routine laboratory inspection methods in human hepatic fibrosis. These two methods were able to efficiently identify advanced hepatic fibrosis stages (F3 and F4) but could not accurately determine the degree of fibrosis for most patients with early (F0 or F1) and intermediate (F2) stages of hepatic fibrosis [34]. Through serum proteomics analysis between the different stages of hepatic fibrosis, differential protein biomarkers were detected between the early, intermediate, and advanced stages and may provide a useful reference for early and intermediate hepatic fibrosis identification. Based on hierarchical clustering and KEGG pathway analysis, the proteomics profile established in the comparison between advanced hepatic fibrosis stages (F3 and F4) showed much greater differences than that in the comparison between early and intermediate stages (control, F1, and F2).

We identified 17 differential serum proteins between healthy volunteers and patients with F1 hepatic fibrosis, and 21 differential proteins were detected between F2 and controls. However, none of the detected differential proteins in these two comparison groups were involved in any pathway. Comparing F1 to F2, 23 differential proteins were analyzed, among which P25 (phospholipid transfer protein (PLTP)) and P34 (lipoprotein (LPA)) were involved in cholesterol metabolism, the only pathway, and both revealed a decreasing trend in the F2 stage. From the analytical data of all differential proteomic profiles of early hepatic fibrosis stages, we concluded that differences exist in the protein level between healthy individuals and patients with F1 or F2 hepatic fibrosis, but significant systematic changes were rarely found between the early stages of hepatic fibrosis. In the comparison between F2 and F3, 41 differential proteins involved in 14 pathways were detected, and between F3 and F4, 46 proteins involved in 19 pathways were found. The analysis in this study confirmed that advanced stages (F3 and F4) were much easier to identify than early stages (F1 and F2) of hepatic fibrosis.

All detected differential proteins may act as potential biomarkers in the comparison between two stages of hepatic fibrosis. For the early stages, no protein exhibited differences concurrently among all three stages (control, F1, and F2). However, the expression abundance of P6 (cartilage intermediate layer protein 2 (CILP2)) and P8 (cilia- and flagella-associated protein 70 (CFAP70)) was differential in healthy people (control) compared with that in F1 and F2 patients. Both proteins showed lower expression abundance in the pathological state than in healthy conditions. Comparing between F1 and F2, lower abundance of P6 and P8 was observed at F2, but the difference in expression was not significant. P6 and P8 may be selected as significant biomarkers to distinguish early stage patients from healthy individuals. CILP-1 has been reported as a novel extracellular matrix protein possessing antifibrotic properties because of its interference of TGF-*β*1 signaling in pressure overload-induced fibrotic remodeling [35]. Yee et al. applied proteomic analysis to explore fibrotic-like changes in degenerate human intervertebral discs and revealed that the expression abundance of CILP and CILP2 was much higher and the mean fibril diameter was smaller in degenerated nucleus pulposus samples of younger individuals [36]. The results demonstrated that CILP2 may also have antifibrotic function. CFAP70, a candidate of cilia-related protein in motile cilia and flagella, has a cluster of tetratricopeptide repeat (TPR) domains. It has been shown that CFAP70 mutations can cause infertility in previous study [37]. In our study, the expression of P6 (CILP2) was much lower in pathological than in healthy situations, and this may be attributed to the similar antifibrotic effect of CILP1. The expression of CFAP70 was much lower in pathological than in healthy situations, and this may be due to cirrhosis reducing the sexual function of the patient [38]. As a result, P6 (CILP2) may be selected as an efficient biomarker to distinguish fibrosis patients from healthy people. For the early stages of F1 and F2, 23 relatively differential proteins were detected. Further investigation might make it possible for us to establish a method of distinguishing between F1 and F2 hepatic fibrosis. P10 (SAA4), P11 (RBP4), and P37 (NCAPH2) expressions were decreased in F2 patients compared to F1 patients, while P23 (HEG1) expression was increased. P10 is a serum amyloid protein that is mainly secreted in plasma and expressed in the liver and declines in the F2 phase probably due to impaired hepatocyte function (https://www.uniprot.org/uniprot/P35542). As a secreted protein of the retinol family, P11 protein is widely distributed in blood, urine, and body fluids. Decreased expression of P11 in F2 may be a new serological indicator reflecting the severity of chronic liver disease and is closely related to the degree of liver tissue fibrosis or cirrhosis [39]. P37 plays a key role in mitotic chromosome assembly and is required for telomerase stabilization. Decreased expression of P37 in F2 may lead to telomere shortening and deletion, which is an important cause of liver fibrosis [40]. P23 is closely associated with tumor progression, and some studies have shown that HEG can promote hepatocarcinogenesis [41]. Therefore, P11 may be selected as a valid biomarker to distinguish F1 and F2 liver fibrosis. Although routine laboratory inspection methods including liver biopsy can accurately identify the F3 and F4 stages of hepatic fibrosis, proteomic analysis data between F3 and F4 should provide an assisted reference for advanced fibrosis.

GO analysis has been widely applied in many areas of research for a better understanding of gene and protein function. Zhao et al. developed a gene function prediction based on GO hierarchy preserving hashing, which was robust in the number of hash functions and was carried out more efficiently than other related methods [42]. Yu et al. found that sequence and GO data associated with protein interaction networks may provide a selectable approach for protein module detection and are helpful in improving performance [43]. In this study, the results of GO analysis revealed that all detected differential proteins between fibrosis stages (F1-vs.-control, F2-vs.-control, F1-vs.-F2, F2-vs.-F3, and F3-vs.-F4) were mainly involved in platelet degranulation and cell adhesion (biological processes), principally played a role in extracellular exosome and extracellular space (cellular components), and exerted an effect on protein binding (molecular function). These findings suggested that the proteins identified herein played a critical role in the pathological immune response.

PPI, a useful tool in proteome analysis, has been utilized in many studies. Karmakar et al. investigated a protein folding system with intricate PPIs to gain new comprehensive research directions using soluble N-ethylmaleimide-sensitive factor attachment receptor complexes, presenting synaptosomal-associated protein 25 as a pathological and diagnostic target [44]. Martin et al. performed a study on PPIs in cancer to develop a screening tool to explore the interactome [45]. Through PPI network analysis, we were able to discover key points from differentially detected proteins. In our study, no differential proteins interacted actively in the PPI network in healthy individuals compared to early fibrosis stages (F1 and F2). In comparing F1 with F2, CALM2 interacted with five proteins and may be selected as a key point for differential analysis. Significant protein interactions existed in the PPI network between F2 and F3 and between F3 and F4. MYH10 (with 6 interacting proteins) and KIF23 (with 5 interacting proteins) were selected for the comparison between F2 and F3. Between F3 and F4, TIE1 (with 12 interacting proteins) and LDHA (with 12 interacting proteins) were detected. All significant protein interactions could be screened out in the differential protein analysis between fibrosis stages. Further investigation on interacting proteins may provide a more accurate and comprehensive analysis of the proteomes between different hepatic fibrosis stages.

## Figures and Tables

**Table 1:** Differential proteome biomarkers identified among early stages of human hepatic fibrosis.

No.	Gene_name	Score	#.Proteins	#.Unique peptides	#.Peptides	*P* value	F1 compared to CON	F2 compared to CON	F1 compared to F2
P1	H2AFJ	0.00000	3	1	1	0.024036062	↓	—	—
P2	KRT1	1101.18878	3	22	26	0.075756699	↓	—	—
P3	ECM1	829.84757	2	20	20	0.070533925	↓	—	—
P4	BLVRB	27.90000	1	1	1	0.094149283	↓	—	—
P5	FLNA	0.00000	3	1	2	0.074369236	↓	—	—
P6	CILP2	32.04	1	1	1	0.09912335	↓	—	—
		32.04000	1	1	1	0.064356888	—	↓	—
P7	SERPINF2	1408.12683	1	25	26	0.052802344	↓	—	—
P8	CFAP70	0.00000	1	1	1	0.072389646	↓	—	—
		0.00000	1	1	1	0.090199296	—	↓	—
P9	OLFM1	85.92000	1	4	4	0.058550382	↑	—	—
P10	SAA4	201.35646	1	10	10	0.006200331	↑	—	—
		201.35646	1	10	10	0.002215994	—	—	↑
P11	RBP4	938.50225	2	23	24	0.034149896	↑	—	—
		938.50225	2	23	24	0.050366617	—	—	↑
P12	CTSZ	25.31000	1	1	1	0.072762411	↑	—	—
P13	TRPV4	30.58000	1	1	1	0.098420076	↑	—	—
P14	FCN2	122.74000	2	5	5	0.022181252	↑	—	—
P15	TRDN	0.00000	1	1	1	0.068395908	↑	—	—
P16	HNRNPCL1	43.77338	7	1	3	0.089950328	↑	—	—
P17	SSC5D	25.92000	4	7	8	0.039565254	↑	—	—
P18	LTF	90.46184	2	6	7	0.06941711	—	—	↓
P19	LRWD1	40.16000	1	1	1	0.072317477	—	—	↓
P20	LGALS3BP	1211.55710	1	23	23	0.073712639	—	—	↓
P21	KRT16	396.14000	19	6	20	0.026026008	—	—	↓
P22	SOD1	36.14000	1	1	1	0.084864486	—	—	↓
P23	HEG1	50.56667	1	3	3	0.026031828	—	—	↓
		50.56667	1	3	3	0.027063278	—	↑	—
P24	LRRC63	0.00000	1	1	1	0.064496929	—	—	↓
P25	PLTP	20.80000	1	4	4	0.025348795	—	—	↑
P26	CTDSP1	0.00000	1	1	1	0.03189535	—	—	↑
P27	CALM2	53.02000	1	1	1	0.034430516	—	—	↑
P28	LMAN2	54.14000	1	1	1	0.025709665	—	—	↑
P29	CAVIN2	17.15000	1	1	1	0.080412212	—	—	↑
P30	ZNHIT6	24.43000	1	1	1	0.076303624	—	—	↑
P31	ZDBF2	0.00000	1	1	1	0.043950877	—	—	↑
P32	STIL	0.00000	1	1	1	0.090414752	—	—	↑
P33	PDLIM5	0.00000	1	1	1	0.064865463	—	—	↑
P34	LPA	247.26695	10	11	15	0.043720414	—	—	↑
P35	ZNF407	0.00000	1	1	1	0.058035207	—	—	↑
P36	TREML1	53.87000	1	1	1	0.045038842	—	—	↑
P37	NCAPH2	0.00000	1	1	1	0.07040615	—	—	↑
		0.00000	1	1	1	0.035792866	—	↓	—
P38	APMAP	111.30000	1	4	4	0.015403975	—	—	↑
P39	DHX29	0.00000	1	1	1	0.074446161	—	↓	—
P40	IGFALS	1063.36355	3	29	29	0.046034759	—	↓	—
P41	RBFA	30.10000	1	1	1	0.084658688	—	↓	—
P42	IL1RAP	96.38968	1	4	4	0.081708039	—	↓	—
P43	SCRIB	42.63000	1	1	1	0.075998794	—	↓	—
P44	SERPINA10	265.07827	1	15	15	0.091672136	—	↓	—
P45	CFL1	50.32000	2	2	2	0.001375553	—	↓	—
P46	LRP1	82.42144	4	8	8	0.069375115	—	↑	—
P47	MCAM	122.44266	1	5	5	0.038473214	—	↑	—
P48	GAN	0.00000	1	1	1	0.030673318	—	↑	—
P49	TGOLN2	61.19000	1	3	3	0.01652702	—	↑	—
P50	UMOD	27.61000	1	2	2	0.07475014	—	↑	—
P51	GPI	0.00000	1	2	2	0.087016827	—	↑	—
P52	SPATA5	0.00000	1	1	1	0.048833958	—	↑	—
P53	IGHA1	484.36598	1	6	10	0.048432175	—	↑	—
P54	CRMP1	0.00000	1	1	1	0.048966322	—	↑	—
P55	ORM2	167.79667	1	3	10	0.068257089	—	↑	—

Differential proteome biomarkers were identified by a series of statistical analysis methods. Gene-name: corresponding gene code for identified protein; Score: matching score by identification; .Proteins: number of detected proteins; Unique.Peptides: number of matched unique peptides; Peptides: number of matched peptides; *P* value: <0.1; F1 compared to CON: expression abundance of detected protein in F1 compared to control. **↑** Upregulated (fold change > 1.2); **↓** downregulated (fold change < 0.8); — no significant difference.

**Table 2:** Differential proteome biomarkers identified between F2 and F3 stage of human hepatic fibrosis.

Gene_name	Score	#.Proteins	#.Unique peptides	#.Peptides	MEAN_F2	MEAN_F3	*P* value	Fold change
CDHR2	67.84000	1	3	3	1.1567284	3.734641735	0.030393796	0.309729415
KIF23	28.40000	1	1	1	1.04066865	1.95383209	0.039067086	0.532629521
ZZEF1	25.17000	1	1	1	0.8163386	1.487453045	0.049786133	0.548816383
CDC45	0.00000	1	1	1	1.12066555	1.869070045	0.084625645	0.599584565
RAD54B	40.41000	1	1	2	0.6076129	0.99280659	0.056997498	0.612015378
LAMC1	0.00000	1	1	1	1.1089689	1.772332385	0.022077332	0.625711582
TTLL8	0.00000	1	1	1	4.05722105	6.31038606	0.079588868	0.642943397
SELP	0.00000	1	1	2	0.724277	1.108942335	0.086915236	0.653124132
CDH5	171.82359	4	7	7	1.24961025	1.77250671	0.003952212	0.704996062
PPIA	47.52000	1	2	2	0.7527572	1.065196395	0.00435389	0.706683954
NCAPH2	0.00000	1	1	1	0.71071275	0.99506203	0.03631808	0.714239644
C9orf72	0.00000	1	1	1	0.9843088	1.353627895	0.043848292	0.727163502
CFD	159.69858	1	4	4	1.11917855	1.5199342	0.079326489	0.736333553
HIST1H4A	34.17000	1	2	2	0.868301	1.16223271	0.077412598	0.747097369
LGALS3BP	1211.55710	1	23	23	0.91597945	1.222727335	0.010768933	0.749128137
FUCA2	71.67000	1	2	2	1.2904989	1.719141185	0.024010826	0.750664873
CD109	70.02583	1	2	4	0.6950532	0.916618035	0.072198557	0.758280083
COL1A1	2125.43260	2	57	60	0.99170045	1.24126761	0.011756652	0.798941696
CAT	268.12000	1	10	10	0.88607235	1.090651505	0.057746831	0.812424818
COL1A2	560.04586	3	31	34	1.00269395	1.232292785	0.07134999	0.813681588
LMAN2	54.14000	1	1	1	1.15866175	1.41990701	0.06521473	0.816012416
COL3A1	33.64000	2	1	3	1.04177725	1.265808275	0.098490075	0.823013461
FABP1	59.87000	1	2	2	0.9981057	1.21195429	0.018034945	0.823550614
TNR	24.25000	1	1	1	0.94406445	0.733598025	0.042108231	1.286896117
CPQ	23.83000	1	1	1	1.00661395	0.77643779	0.002213724	1.296451516
IL1RAP	96.38968	1	4	4	0.79807365	0.604451735	0.096536441	1.32032651
UMOD	27.61000	1	2	2	1.1963501	0.89455069	0.010180284	1.337375415
GNPTG	0.00000	1	2	2	1.3583153	1.00752861	0.054291649	1.348165488
CTBS	35.04000	1	3	3	0.85170885	0.629964875	0.083385639	1.351994189
C4A	9942.66108	9	5	179	1.0382126	0.72739336	0.046765794	1.427305578
CNDP1	489.80074	1	16	16	1.0953562	0.726863725	0.088400653	1.506962258
APCS	670.46990	1	9	9	1.0782359	0.625951395	0.092351646	1.722555311
MGA	0.00000	1	1	1	0.8007978	0.464695475	0.050165383	1.723274366
LTF	90.46184	2	6	7	1.31309015	0.7046773	0.074555121	1.863392151
ANKHD1	27.69000	2	1	2	0.84187635	0.43930618	0.090521544	1.916377206
LRWD1	40.16000	1	1	1	1.25997445	0.529221705	0.005758134	2.380806452
ORM2	167.79667	1	3	10	1.91838385	0.796487075	0.085053393	2.408556159
MYH10	0.00000	1	1	1	0.5197956	0.20681813	0.014314988	2.513298036
SERPINE1	54.42000	1	1	1	0.7277185	0.28168128	0.013278244	2.583481941
ATXN2	0.00000	1	1	1	0.51939755	0.13630007	0.086563626	3.810691733
TUFM	0.00000	1	1	1	0.44915935	0.09972096	0.094500863	4.504161913

Differential proteome biomarkers were identified by a series of statistical analysis methods. Gene-name: corresponding gene code for identified protein; Score: matching score by identification; .Proteins: number of detected proteins; Unique.Peptides: number of matched unique peptides; Peptides: number of matched peptides; *P* value: <0.1; MEAN-F2: average expression abundance of two biological replicates in F2 group; MEAN-F3: average expression abundance of two biological replicates in F3 group. Upregulated (fold change > 1.2); downregulated (fold change < 0.8).

**Table 3:** Differential proteome biomarkers identified between F3 and F4 stage of human hepatic fibrosis.

Gene_name	Score	#.Proteins	#.Unique peptides	#.Peptides	MEAN_F3	MEAN_F4	*P* value	Fold change
HIST1H2BK	58.78788	18	3	4	0.91694111	4.567607215	0.02275837	0.200748678
PSAP	178.06571	1	2	2	2.21498952	9.866392995	0.081923017	0.224498408
HIST1H3A	30.10000	3	1	1	1.007118065	3.450025825	0.055961473	0.291916095
SERPINE1	54.42000	1	1	1	0.28168128	0.801853925	0.01566317	0.351287524
TALDO1	25.87000	1	1	1	1.1134471	2.436683265	0.01653216	0.456951921
ADRA1D	0.00000	1	1	1	0.448665155	0.90547077	0.040495495	0.495504847
GPI	0.00000	1	2	2	1.136271275	2.27594925	0.047438887	0.499251587
IGLV1-51	49.24000	2	2	2	0.72031513	1.41407929	0.023970467	0.509388077
LDHA	182.13500	3	5	7	0.98430261	1.74136361	0.026047479	0.565248179
JCHAIN	0.00000	1	1	1	0.775689805	1.297338645	0.064848068	0.597908501
CORO1A	52.96000	1	1	1	1.02980141	1.699410955	0.003867435	0.605975504
YWHAZ	53.67000	3	2	2	1.329026125	2.18042104	0.059619436	0.609527289
VASP	21.22000	1	1	1	1.18331832	1.935903255	0.001346437	0.611248686
MGA	0.00000	1	1	1	0.464695475	0.75144601	0.023766465	0.61840168
TPM3	47.11000	3	1	2	1.07130211	1.66324595	0.06686543	0.644103243
SOD3	55.11000	1	3	3	0.349465775	0.529788505	0.013630205	0.659632611
LCP1	264.31215	3	15	16	0.94516355	1.427876255	0.076290676	0.661936598
LSAMP	31.17000	1	2	2	1.10493157	1.64859985	0.097795998	0.670224233
OR5A2	28.86000	1	1	1	0.52274932	0.7628278	0.066462212	0.685278276
TRDN	0.00000	1	1	1	1.389876265	1.974410795	0.069478636	0.703944827
TIE1	40.70000	1	2	2	1.103988345	1.51950628	0.041233163	0.726544115
CDH2	0.00000	1	2	2	1.199460695	1.59554698	0.038173559	0.751755172
CCDC137	0.00000	1	1	1	0.815490835	1.07219904	0.081920717	0.760577845
LARS	0.00000	1	2	2	1.0754855	1.33752222	0.020995556	0.80408795
AHSG	6661.90805	1	47	47	0.956198385	0.752478425	0.02549769	1.270731962
CDH5	171.82359	4	7	7	1.77250671	1.38291291	0.038095089	1.281719693
FGL2	0.00000	1	1	1	1.271405835	0.98971743	0.059754514	1.284614978
CARD6	0.00000	1	1	1	0.75718159	0.58356132	0.076205764	1.297518468
SERPINF2	1408.12683	1	25	26	0.92664857	0.710921615	0.082596355	1.303446893
IL6ST	24.33000	1	1	1	1.46089552	1.09962861	0.055202499	1.328535386
SERPING1	3869.86349	2	49	49	1.05476914	0.788823005	0.041773876	1.337142975
PARD3	0.00000	1	1	1	0.825738295	0.609493725	0.099038026	1.354793759
PODXL	0.00000	1	2	2	1.4057577	1.028178575	0.091751521	1.367231077
KNG1	8235.22033	1	89	90	1.04430991	0.75566476	0.09603675	1.381975137
CDKL1	0.00000	2	1	1	1.25846535	0.85403005	0.098225782	1.473560971
FUCA2	71.67000	1	2	2	1.719141185	1.132890385	0.015356564	1.517482369
CADM1	93.80667	1	1	1	1.30906101	0.843302865	0.097176886	1.552302339
CFD	159.69858	1	4	4	1.5199342	0.93293038	0.060805301	1.629204314
IGHG4	234.37444	1	1	6	1.206264895	0.680698885	0.066048639	1.772097651
PSMC2	60.25782	9	1	2	0.8917285	0.494511585	0.033185676	1.803250979
RPGR	0.00000	1	1	1	0.718604715	0.382276295	0.044101616	1.87980454
CFHR1	573.43387	1	3	18	1.124351305	0.57180219	0.043641926	1.966329134
TBX18	0.00000	1	1	1	0.60035456	0.288173085	0.072236041	2.083312395
CDHR2	67.84000	1	3	3	3.734641735	1.776737215	0.096422533	2.101966292
ZNF407	0.00000	1	1	1	0.455257525	0.17873621	0.07633917	2.547091745
ATXN2	0.00000	1	1	1	0.13630007	0.03008251	0.079890987	4.530874252

Differential proteome biomarkers were identified by a series of statistical analysis methods. Gene-name: corresponding gene code for identified protein; Score: matching score by identification; .Proteins: number of detected proteins; Unique.Peptides: number of matched unique peptides; Peptides: number of matched peptides; *P* value: <0.1; MEAN-F3: average expression abundance of two biological replicates in F3 group; MEAN-F4: average expression abundance of two biological replicates in F4 group. Upregulated (fold change > 1.2); downregulated (fold change < 0.8).

## Data Availability

The data used to support the findings of this study are available from the corresponding author upon request.

## Supplementary Materials

Supplementary Materials Table S1: the descriptions of the differential proteome. Table S2: differential proteins between F1 and CON stage of hepatic fibrosis. Table S3: differential proteins between F2 and CON stage of hepatic fibrosis. Table S4: differential proteins between F1 and F2 stage of hepatic fibrosis. Table S5: differential proteins between F2 and F3 stage of hepatic fibrosis. Table S6: differential proteins between F3 and F4 stage of hepatic fibrosis. Table S7: KEGG pathway analysis of the identified differential proteins of hepatic fibrosis. Table S8: PPI network analysis between hepatic fibrosis stages. Figure S1: functional annotation of proteins with GO between F2 and F3 stage of hepatic fibrosis. Figure S2: functional annotation of proteins with GO between F3 and F4 stage of hepatic fibrosis. Figure S3: PPI of differentially expressed proteins between F2 and F3 stage of hepatic fibrosis. Figure S4: PPI of differentially expressed proteins between F3 and F4 stage of hepatic fibrosis.
