# Supplementary material for: Proteomic Analysis of Human Serum for Patients at Different Pathological Stages of Hepatic Fibrosis
Source: Biomed Res Int. 2021 Nov 28;2021:3580090. doi: 10.1155/2021/3580090 (PMC8645358; doi:10.1155/2021/3580090)

**Table S1 The descriptions of the differential proteome**

| Gene_name | Accession | Description |
| --- | --- | --- |
| H2AFJ | Q9BTM1 | Histone H2A.J OS=Homo sapiens GN=H2AFJ PE=1 SV=1 - [H2AJ_HUMAN] |
| KRT1 | P04264 | Keratin, type II cytoskeletal 1 OS=Homo sapiens GN=KRT1 PE=1 SV=6 - [K2C1_HUMAN] |
| ECM1 | Q16610 | Extracellular matrix protein 1 OS=Homo sapiens GN=ECM1 PE=1 SV=2 - [ECM1_HUMAN] |
| BLVRB | P30043 | Flavin reductase (NADPH) OS=Homo sapiens GN=BLVRB PE=1 SV=3 - [BLVRB_HUMAN] |
| FLNA | P21333 | Filamin-A OS=Homo sapiens GN=FLNA PE=1 SV=4 - [FLNA_HUMAN] |
| CILP2 | Q8IUL8 | Cartilage intermediate layer protein 2 OS=Homo sapiens GN=CILP2 PE=2 SV=2 - [CILP2_HUMAN] |
| SERPINF2 | P08697 | Alpha-2-antiplasmin OS=Homo sapiens GN=SERPINF2 PE=1 SV=3 - [A2AP_HUMAN] |
| CFAP70 | Q5T0N1 | Cilia- and flagella-associated protein 70 OS=Homo sapiens GN=CFAP70 PE=2 SV=3 - [CFA70_HUMAN] |
| OLFM1 | Q99784 | Noelin OS=Homo sapiens GN=OLFM1 PE=1 SV=4 - [NOE1_HUMAN] |
| SAA4 | P35542 | Serum amyloid A-4 protein OS=Homo sapiens GN=SAA4 PE=1 SV=2 - [SAA4_HUMAN] |
| RBP4 | P02753 | Retinol-binding protein 4 OS=Homo sapiens GN=RBP4 PE=1 SV=3 - [RET4_HUMAN] |
| CTSZ | Q9UBR2 | Cathepsin Z OS=Homo sapiens GN=CTSZ PE=1 SV=1 - [CATZ_HUMAN] |
| TRPV4 | Q9HBA0 | Transient receptor potential cation channel subfamily V member 4 OS=Homo sapiens GN=TRPV4 PE=1 SV=2 - [TRPV4_HUMAN] |
| FCN2 | Q15485 | Ficolin-2 OS=Homo sapiens GN=FCN2 PE=1 SV=2 - [FCN2_HUMAN] |
| TRDN | Q13061 | Triadin OS=Homo sapiens GN=TRDN PE=1 SV=4 - [TRDN_HUMAN] |
| HNRNPCL1 | O60812 | Heterogeneous nuclear ribonucleoprotein C-like 1 OS=Homo sapiens GN=HNRNPCL1 PE=1 SV=1 - [HNRC1_HUMAN] |
| SSC5D | A1L4H1 | Soluble scavenger receptor cysteine-rich domain-containing protein SSC5D OS=Homo sapiens GN=SSC5D PE=1 SV=3 - [SRCRL_HUMAN] |
| LTF | P02788 | Lactotransferrin OS=Homo sapiens GN=LTF PE=1 SV=6 - [TRFL_HUMAN] |
| LRWD1 | Q9UFC0 | Leucine-rich repeat and WD repeat-containing protein 1 OS=Homo sapiens GN=LRWD1 PE=1 SV=2 - [LRWD1_HUMAN] |
| LGALS3BP | Q08380 | Galectin-3-binding protein OS=Homo sapiens GN=LGALS3BP PE=1 SV=1 - [LG3BP_HUMAN] |
| KRT16 | P08779 | Keratin, type I cytoskeletal 16 OS=Homo sapiens GN=KRT16 PE=1 SV=4 - [K1C16_HUMAN] |
| SOD1 | P00441 | Superoxide dismutase [Cu-Zn] OS=Homo sapiens GN=SOD1 PE=1 SV=2 - [SODC_HUMAN] |
| HEG1 | Q9ULI3 | Protein HEG homolog 1 OS=Homo sapiens GN=HEG1 PE=1 SV=3 - [HEG1_HUMAN] |
| LRRC63 | Q05C16 | Leucine-rich repeat-containing protein 63 OS=Homo sapiens GN=LRRC63 PE=2 SV=2 - [LRC63_HUMAN] |
| PLTP | P55058 | Phospholipid transfer protein OS=Homo sapiens GN=PLTP PE=1 SV=1 - [PLTP_HUMAN] |
| CTDSP1 | Q9GZU7 | Carboxy-terminal domain RNA polymerase II polypeptide A small phosphatase 1 OS=Homo sapiens GN=CTDSP1 PE=1 SV=1 - [CTDS1_HUMAN] |
| CALM2 | P0DP24 | Calmodulin-2 OS=Homo sapiens GN=CALM2 PE=1 SV=1 - [CALM2_HUMAN] |
| LMAN2 | Q12907 | Vesicular integral-membrane protein VIP36 OS=Homo sapiens GN=LMAN2 PE=1 SV=1 - [LMAN2_HUMAN] |
| CAVIN2 | O95810 | Caveolae-associated protein 2 OS=Homo sapiens GN=CAVIN2 PE=1 SV=3 - [CAVN2_HUMAN] |
| ZNHIT6 | Q9NWK9 | Box C/D snoRNA protein 1 OS=Homo sapiens GN=ZNHIT6 PE=1 SV=1 - [BCD1_HUMAN] |
| ZDBF2 | Q9HCK1 | DBF4-type zinc finger-containing protein 2 OS=Homo sapiens GN=ZDBF2 PE=1 SV=3 - [ZDBF2_HUMAN] |
| STIL | Q15468 | SCL-interrupting locus protein OS=Homo sapiens GN=STIL PE=1 SV=2 - [STIL_HUMAN] |
| PDLIM5 | Q96HC4 | PDZ and LIM domain protein 5 OS=Homo sapiens GN=PDLIM5 PE=1 SV=5 - [PDLI5_HUMAN] |
| LPA | P08519 | Apolipoprotein(a) OS=Homo sapiens GN=LPA PE=1 SV=1 - [APOA_HUMAN] |
| ZNF407 | Q9C0G0 | Zinc finger protein 407 OS=Homo sapiens GN=ZNF407 PE=1 SV=2 - [ZN407_HUMAN] |
| TREML1 | Q86YW5 | Trem-like transcript 1 protein OS=Homo sapiens GN=TREML1 PE=1 SV=2 - [TRML1_HUMAN] |
| NCAPH2 | Q6IBW4 | Condensin-2 complex subunit H2 OS=Homo sapiens GN=NCAPH2 PE=1 SV=1 - [CNDH2_HUMAN] |
| APMAP | Q9HDC9 | Adipocyte plasma membrane-associated protein OS=Homo sapiens GN=APMAP PE=1 SV=2 - [APMAP_HUMAN] |
| DHX29 | Q7Z478 | ATP-dependent RNA helicase DHX29 OS=Homo sapiens GN=DHX29 PE=1 SV=2 - [DHX29_HUMAN] |
| IGFALS | P35858 | Insulin-like growth factor-binding protein complex acid labile subunit OS=Homo sapiens GN=IGFALS PE=1 SV=1 - [ALS_HUMAN] |
| RBFA | Q8N0V3 | Putative ribosome-binding factor A, mitochondrial OS=Homo sapiens GN=RBFA PE=1 SV=3 - [RBFA_HUMAN] |
| IL1RAP | Q9NPH3 | Interleukin-1 receptor accessory protein OS=Homo sapiens GN=IL1RAP PE=1 SV=2 - [IL1AP_HUMAN] |
| SCRIB | Q14160 | Protein scribble homolog OS=Homo sapiens GN=SCRIB PE=1 SV=4 - [SCRIB_HUMAN] |
| SERPINA10 | Q9UK55 | Protein Z-dependent protease inhibitor OS=Homo sapiens GN=SERPINA10 PE=1 SV=1 - [ZPI_HUMAN] |
| CFL1 | P23528 | Cofilin-1 OS=Homo sapiens GN=CFL1 PE=1 SV=3 - [COF1_HUMAN] |
| LRP1 | Q07954 | Prolow-density lipoprotein receptor-related protein 1 OS=Homo sapiens GN=LRP1 PE=1 SV=2 - [LRP1_HUMAN] |
| MCAM | P43121 | Cell surface glycoprotein MUC18 OS=Homo sapiens GN=MCAM PE=1 SV=2 - [MUC18_HUMAN] |
| GAN | Q9H2C0 | Gigaxonin OS=Homo sapiens GN=GAN PE=1 SV=1 - [GAN_HUMAN] |
| TGOLN2 | O43493 | Trans-Golgi network integral membrane protein 2 OS=Homo sapiens GN=TGOLN2 PE=1 SV=2 - [TGON2_HUMAN] |
| UMOD | P07911 | Uromodulin OS=Homo sapiens GN=UMOD PE=1 SV=1 - [UROM_HUMAN] |
| GPI | P06744 | Glucose-6-phosphate isomerase OS=Homo sapiens GN=GPI PE=1 SV=4 - [G6PI_HUMAN] |
| SPATA5 | Q8NB90 | Spermatogenesis-associated protein 5 OS=Homo sapiens GN=SPATA5 PE=1 SV=3 - [SPAT5_HUMAN] |
| IGHA1 | P01876 | Immunoglobulin heavy constant alpha 1 OS=Homo sapiens GN=IGHA1 PE=1 SV=2 - [IGHA1_HUMAN] |
| CRMP1 | Q14194 | Dihydropyrimidinase-related protein 1 OS=Homo sapiens GN=CRMP1 PE=1 SV=1 - [DPYL1_HUMAN] |
| ORM2 | P19652 | Alpha-1-acid glycoprotein 2 OS=Homo sapiens GN=ORM2 PE=1 SV=2 - [A1AG2_HUMAN] |
| CDHR2 | Q9BYE9 | Cadherin-related family member 2 OS=Homo sapiens GN=CDHR2 PE=1 SV=2 - [CDHR2_HUMAN] |
| KIF23 | Q02241 | Kinesin-like protein KIF23 OS=Homo sapiens GN=KIF23 PE=1 SV=3 - [KIF23_HUMAN] |
| ZZEF1 | O43149 | Zinc finger ZZ-type and EF-hand domain-containing protein 1 OS=Homo sapiens GN=ZZEF1 PE=1 SV=6 - [ZZEF1_HUMAN] |
| CDC45 | O75419 | Cell division control protein 45 homolog OS=Homo sapiens GN=CDC45 PE=1 SV=1 - [CDC45_HUMAN] |
| RAD54B | Q9Y620 | DNA repair and recombination protein RAD54B OS=Homo sapiens GN=RAD54B PE=1 SV=1 - [RA54B_HUMAN] |
| LAMC1 | P11047 | Laminin subunit gamma-1 OS=Homo sapiens GN=LAMC1 PE=1 SV=3 - [LAMC1_HUMAN] |
| TTLL8 | A6PVC2 | Protein monoglycylase TTLL8 OS=Homo sapiens GN=TTLL8 PE=2 SV=4 - [TTLL8_HUMAN] |
| SELP | P16109 | P-selectin OS=Homo sapiens GN=SELP PE=1 SV=3 - [LYAM3_HUMAN] |
| CDH5 | P33151 | Cadherin-5 OS=Homo sapiens GN=CDH5 PE=1 SV=5 - [CADH5_HUMAN] |
| PPIA | P62937 | Peptidyl-prolyl cis-trans isomerase A OS=Homo sapiens GN=PPIA PE=1 SV=2 - [PPIA_HUMAN] |
| NCAPH2 | Q6IBW4 | Condensin-2 complex subunit H2 OS=Homo sapiens GN=NCAPH2 PE=1 SV=1 - [CNDH2_HUMAN] |
| C9orf72 | Q96LT7 | Guanine nucleotide exchange C9orf72 OS=Homo sapiens GN=C9orf72 PE=1 SV=2 - [CI072_HUMAN] |
| CFD | P00746 | Complement factor D OS=Homo sapiens GN=CFD PE=1 SV=5 - [CFAD_HUMAN] |
| HIST1H4A | P62805 | Histone H4 OS=Homo sapiens GN=HIST1H4A PE=1 SV=2 - [H4_HUMAN] |
| LGALS3BP | Q08380 | Galectin-3-binding protein OS=Homo sapiens GN=LGALS3BP PE=1 SV=1 - [LG3BP_HUMAN] |
| FUCA2 | Q9BTY2 | Plasma alpha-L-fucosidase OS=Homo sapiens GN=FUCA2 PE=1 SV=2 - [FUCO2_HUMAN] |
| CD109 | Q6YHK3 | CD109 antigen OS=Homo sapiens GN=CD109 PE=1 SV=2 - [CD109_HUMAN] |
| COL1A1 | P02452 | Collagen alpha-1(I) chain OS=Homo sapiens GN=COL1A1 PE=1 SV=5 - [CO1A1_HUMAN] |
| CAT | P04040 | Catalase OS=Homo sapiens GN=CAT PE=1 SV=3 - [CATA_HUMAN] |
| COL1A2 | P08123 | Collagen alpha-2(I) chain OS=Homo sapiens GN=COL1A2 PE=1 SV=7 - [CO1A2_HUMAN] |
| LMAN2 | Q12907 | Vesicular integral-membrane protein VIP36 OS=Homo sapiens GN=LMAN2 PE=1 SV=1 - [LMAN2_HUMAN] |
| COL3A1 | P02461 | Collagen alpha-1(III) chain OS=Homo sapiens GN=COL3A1 PE=1 SV=4 - [CO3A1_HUMAN] |
| FABP1 | P07148 | Fatty acid-binding protein, liver OS=Homo sapiens GN=FABP1 PE=1 SV=1 - [FABPL_HUMAN] |
| TNR | Q92752 | Tenascin-R OS=Homo sapiens GN=TNR PE=1 SV=3 - [TENR_HUMAN] |
| CPQ | Q9Y646 | Carboxypeptidase Q OS=Homo sapiens GN=CPQ PE=1 SV=1 - [CBPQ_HUMAN] |
| IL1RAP | Q9NPH3 | Interleukin-1 receptor accessory protein OS=Homo sapiens GN=IL1RAP PE=1 SV=2 - [IL1AP_HUMAN] |
| UMOD | P07911 | Uromodulin OS=Homo sapiens GN=UMOD PE=1 SV=1 - [UROM_HUMAN] |
| GNPTG | Q9UJJ9 | N-acetylglucosamine-1-phosphotransferase subunit gamma OS=Homo sapiens GN=GNPTG PE=1 SV=1 - [GNPTG_HUMAN] |
| CTBS | Q01459 | Di-N-acetylchitobiase OS=Homo sapiens GN=CTBS PE=1 SV=1 - [DIAC_HUMAN] |
| C4A | P0C0L4 | Complement C4-A OS=Homo sapiens GN=C4A PE=1 SV=2 - [CO4A_HUMAN] |
| CNDP1 | Q96KN2 | Beta-Ala-His dipeptidase OS=Homo sapiens GN=CNDP1 PE=1 SV=4 - [CNDP1_HUMAN] |
| APCS | P02743 | Serum amyloid P-component OS=Homo sapiens GN=APCS PE=1 SV=2 - [SAMP_HUMAN] |
| MGA | Q8IWI9 | MAX gene-associated protein OS=Homo sapiens GN=MGA PE=1 SV=3 - [MGAP_HUMAN] |
| LTF | P02788 | Lactotransferrin OS=Homo sapiens GN=LTF PE=1 SV=6 - [TRFL_HUMAN] |
| ANKHD1 | Q8IWZ3 | Ankyrin repeat and KH domain-containing protein 1 OS=Homo sapiens GN=ANKHD1 PE=1 SV=1 - [ANKH1_HUMAN] |
| LRWD1 | Q9UFC0 | Leucine-rich repeat and WD repeat-containing protein 1 OS=Homo sapiens GN=LRWD1 PE=1 SV=2 - [LRWD1_HUMAN] |
| ORM2 | P19652 | Alpha-1-acid glycoprotein 2 OS=Homo sapiens GN=ORM2 PE=1 SV=2 - [A1AG2_HUMAN] |
| MYH10 | P35580 | Myosin-10 OS=Homo sapiens GN=MYH10 PE=1 SV=3 - [MYH10_HUMAN] |
| SERPINE1 | P05121 | Plasminogen activator inhibitor 1 OS=Homo sapiens GN=SERPINE1 PE=1 SV=1 - [PAI1_HUMAN] |
| ATXN2 | Q99700 | Ataxin-2 OS=Homo sapiens GN=ATXN2 PE=1 SV=2 - [ATX2_HUMAN] |
| TUFM | P49411 | Elongation factor Tu, mitochondrial OS=Homo sapiens GN=TUFM PE=1 SV=2 - [EFTU_HUMAN] |
| HIST1H2BK | O60814 | Histone H2B type 1-K OS=Homo sapiens GN=HIST1H2BK PE=1 SV=3 - [H2B1K_HUMAN] |
| PSAP | P07602 | Prosaposin OS=Homo sapiens GN=PSAP PE=1 SV=2 - [SAP_HUMAN] |
| HIST1H3A | P68431 | Histone H3.1 OS=Homo sapiens GN=HIST1H3A PE=1 SV=2 - [H31_HUMAN] |
| SERPINE1 | P05121 | Plasminogen activator inhibitor 1 OS=Homo sapiens GN=SERPINE1 PE=1 SV=1 - [PAI1_HUMAN] |
| TALDO1 | P37837 | Transaldolase OS=Homo sapiens GN=TALDO1 PE=1 SV=2 - [TALDO_HUMAN] |
| ADRA1D | P25100 | Alpha-1D adrenergic receptor OS=Homo sapiens GN=ADRA1D PE=1 SV=2 - [ADA1D_HUMAN] |
| GPI | P06744 | Glucose-6-phosphate isomerase OS=Homo sapiens GN=GPI PE=1 SV=4 - [G6PI_HUMAN] |
| IGLV1-51 | P01701 | Immunoglobulin lambda variable 1-51 OS=Homo sapiens GN=IGLV1-51 PE=1 SV=2 - [LV151_HUMAN] |
| LDHA | P00338 | L-lactate dehydrogenase A chain OS=Homo sapiens GN=LDHA PE=1 SV=2 - [LDHA_HUMAN] |
| JCHAIN | P01591 | Immunoglobulin J chain OS=Homo sapiens GN=JCHAIN PE=1 SV=4 - [IGJ_HUMAN] |
| CORO1A | P31146 | Coronin-1A OS=Homo sapiens GN=CORO1A PE=1 SV=4 - [COR1A_HUMAN] |
| YWHAZ | P63104 | 14-3-3 protein zeta/delta OS=Homo sapiens GN=YWHAZ PE=1 SV=1 - [1433Z_HUMAN] |
| VASP | P50552 | Vasodilator-stimulated phosphoprotein OS=Homo sapiens GN=VASP PE=1 SV=3 - [VASP_HUMAN] |
| MGA | Q8IWI9 | MAX gene-associated protein OS=Homo sapiens GN=MGA PE=1 SV=3 - [MGAP_HUMAN] |
| TPM3 | P06753 | Tropomyosin alpha-3 chain OS=Homo sapiens GN=TPM3 PE=1 SV=2 - [TPM3_HUMAN] |
| SOD3 | P08294 | Extracellular superoxide dismutase [Cu-Zn] OS=Homo sapiens GN=SOD3 PE=1 SV=2 - [SODE_HUMAN] |
| LCP1 | P13796 | Plastin-2 OS=Homo sapiens GN=LCP1 PE=1 SV=6 - [PLSL_HUMAN] |
| LSAMP | Q13449 | Limbic system-associated membrane protein OS=Homo sapiens GN=LSAMP PE=1 SV=2 - [LSAMP_HUMAN] |
| OR5A2 | Q8NGI9 | Olfactory receptor 5A2 OS=Homo sapiens GN=OR5A2 PE=2 SV=1 - [OR5A2_HUMAN] |
| TRDN | Q13061 | Triadin OS=Homo sapiens GN=TRDN PE=1 SV=4 - [TRDN_HUMAN] |
| TIE1 | P35590 | Tyrosine-protein kinase receptor Tie-1 OS=Homo sapiens GN=TIE1 PE=1 SV=1 - [TIE1_HUMAN] |
| CDH2 | P19022 | Cadherin-2 OS=Homo sapiens GN=CDH2 PE=1 SV=4 - [CADH2_HUMAN] |
| CCDC137 | Q6PK04 | Coiled-coil domain-containing protein 137 OS=Homo sapiens GN=CCDC137 PE=1 SV=1 - [CC137_HUMAN] |
| LARS | Q9P2J5 | Leucine--tRNA ligase, cytoplasmic OS=Homo sapiens GN=LARS PE=1 SV=2 - [SYLC_HUMAN] |
| AHSG | P02765 | Alpha-2-HS-glycoprotein OS=Homo sapiens GN=AHSG PE=1 SV=1 - [FETUA_HUMAN] |
| CDH5 | P33151 | Cadherin-5 OS=Homo sapiens GN=CDH5 PE=1 SV=5 - [CADH5_HUMAN] |
| FGL2 | Q14314 | Fibroleukin OS=Homo sapiens GN=FGL2 PE=1 SV=1 - [FGL2_HUMAN] |
| CARD6 | Q9BX69 | Caspase recruitment domain-containing protein 6 OS=Homo sapiens GN=CARD6 PE=1 SV=2 - [CARD6_HUMAN] |
| SERPINF2 | P08697 | Alpha-2-antiplasmin OS=Homo sapiens GN=SERPINF2 PE=1 SV=3 - [A2AP_HUMAN] |
| IL6ST | P40189 | Interleukin-6 receptor subunit beta OS=Homo sapiens GN=IL6ST PE=1 SV=2 - [IL6RB_HUMAN] |
| SERPING1 | P05155 | Plasma protease C1 inhibitor OS=Homo sapiens GN=SERPING1 PE=1 SV=2 - [IC1_HUMAN] |
| PARD3 | Q8TEW0 | Partitioning defective 3 homolog OS=Homo sapiens GN=PARD3 PE=1 SV=2 - [PARD3_HUMAN] |
| PODXL | O00592 | Podocalyxin OS=Homo sapiens GN=PODXL PE=1 SV=2 - [PODXL_HUMAN] |
| KNG1 | P01042 | Kininogen-1 OS=Homo sapiens GN=KNG1 PE=1 SV=2 - [KNG1_HUMAN] |
| CDKL1 | Q00532 | Cyclin-dependent kinase-like 1 OS=Homo sapiens GN=CDKL1 PE=1 SV=5 - [CDKL1_HUMAN] |
| FUCA2 | Q9BTY2 | Plasma alpha-L-fucosidase OS=Homo sapiens GN=FUCA2 PE=1 SV=2 - [FUCO2_HUMAN] |
| CADM1 | Q9BY67 | Cell adhesion molecule 1 OS=Homo sapiens GN=CADM1 PE=1 SV=2 - [CADM1_HUMAN] |
| CFD | P00746 | Complement factor D OS=Homo sapiens GN=CFD PE=1 SV=5 - [CFAD_HUMAN] |
| IGHG4 | P01861 | Immunoglobulin heavy constant gamma 4 OS=Homo sapiens GN=IGHG4 PE=1 SV=1 - [IGHG4_HUMAN] |
| PSMC2 | P35998 | 26S proteasome regulatory subunit 7 OS=Homo sapiens GN=PSMC2 PE=1 SV=3 - [PRS7_HUMAN] |
| RPGR | Q92834 | X-linked retinitis pigmentosa GTPase regulator OS=Homo sapiens GN=RPGR PE=1 SV=2 - [RPGR_HUMAN] |
| CFHR1 | Q03591 | Complement factor H-related protein 1 OS=Homo sapiens GN=CFHR1 PE=1 SV=2 - [FHR1_HUMAN] |
| TBX18 | O95935 | T-box transcription factor TBX18 OS=Homo sapiens GN=TBX18 PE=1 SV=3 - [TBX18_HUMAN] |
| CDHR2 | Q9BYE9 | Cadherin-related family member 2 OS=Homo sapiens GN=CDHR2 PE=1 SV=2 - [CDHR2_HUMAN] |
| ZNF407 | Q9C0G0 | Zinc finger protein 407 OS=Homo sapiens GN=ZNF407 PE=1 SV=2 - [ZN407_HUMAN] |

**Gene-name**: corresponding gene code for identified protein; **Description**: description of detected proteins.

**TableS2 Differential proteins between F1 and CON stage of hepatic fibrosis**.

|  | Term | Count | % | PValue | Genes |
| --- | --- | --- | --- | --- | --- |
| B  P | GO:0002576~platelet degranulation | 3 | 0.148957 | 2.78E-03 | SERPINF2, ECM1, FLNA |
|  | GO:0001867~complement activation, lectin pathway | 2 | 0.099305 | 5.41E-03 | FCN2, KRT1 |
|  | GO:0042730~fibrinolysis | 2 | 0.099305 | 1.61E-02 | SERPINF2, KRT1 |
|  | GO:0006953~acute-phase response | 2 | 0.099305 | 2.98E-02 | SERPINF2, SAA4 |
|  | GO:0031532~actin cytoskeleton reorganization | 2 | 0.099305 | 3.58E-02 | TRPV4, FLNA |
|  | GO:0050829~defense response to Gram-negative bacterium | 2 | 0.099305 | 4.18E-02 | FCN2, SSC5D |
|  | GO:0046330~positive regulation of JNK cascade | 2 | 0.099305 | 4.92E-02 | SERPINF2, TRPV4 |
|  | GO:0050830~defense response to Gram-positive bacterium | 2 | 0.099305 | 6.39E-02 | FCN2, SSC5D |
|  | GO:0006874~cellular calcium ion homeostasis | 2 | 0.099305 | 6.97E-02 | TRDN, TRPV4 |
|  | GO:0042593~glucose homeostasis | 2 | 0.099305 | 7.55E-02 | RBP4, TRPV4 |
| C  C | GO:0070062~extracellular exosome | 12 | 0.595829 | 2.37E-06 | CFAP70, RBP4, CTSZ, CILP2, SERPINF2, FCN2, BLVRB, KRT1, SAA4, H2AFJ, ECM1, FLNA |
|  | GO:0005615~extracellular space | 8 | 0.397219 | 7.49E-05 | RBP4, CTSZ, SERPINF2, KRT1, SAA4, ECM1, SSC5D, OLFM1 |
|  | GO:0031012~extracellular matrix | 4 | 0.19861 | 2.03E-03 | KRT1, ECM1, SSC5D, FLNA |
|  | GO:0072562~blood microparticle | 3 | 0.148957 | 7.68E-03 | SERPINF2, FCN2, KRT1 |
|  | GO:0005576~extracellular region | 6 | 0.297915 | 1.02E-02 | RBP4, SERPINF2, FCN2, SAA4, ECM1, FLNA |
|  | GO:0005929~cilium | 2 | 0.099305 | 1.25E-01 | CFAP70, TRPV4 |
|  | GO:0005783~endoplasmic reticulum | 3 | 0.148957 | 1.63E-01 | TRDN, CTSZ, OLFM1 |
|  | GO:0005578~proteinaceous extracellular matrix | 2 | 0.099305 | 0.211119 | CILP2, ECM1 |
|  | GO:0043025~neuronal cell body | 2 | 0.099305 | 0.24353 | FLNA, OLFM1 |
|  | GO:0005886~plasma membrane | 6 | 0.297915 | 0.285789 | TRDN, CTSZ, BLVRB, KRT1, TRPV4, FLNA |
| M  F | GO:0043236~laminin binding | 2 | 0.099305 | 2.20E-02 | ECM1, SSC5D |
|  | GO:0002020~protease binding | 2 | 0.099305 | 8.61E-02 | SERPINF2, ECM1 |
|  | GO:0044325~ion channel binding | 2 | 0.099305 | 9.59E-02 | TRDN, FLNA |
|  | GO:0051015~actin filament binding | 2 | 0.099305 | 1.11E-01 | TRPV4, FLNA |
|  | GO:0004871~signal transducer activity | 2 | 0.099305 | 1.67E-01 | ECM1, FLNA |
|  | GO:0005515~protein binding | 11 | 0.546177 | 0.191239 | TRDN, RBP4, CTSZ, SERPINF2, FCN2, KRT1, TRPV4, ECM1, SSC5D, FLNA, OLFM1 |
|  | GO:0046982~protein heterodimerization activity | 2 | 0.099305 | 0.342401 | RBP4, H2AFJ |
|  | GO:0042803~protein homodimerization activity | 2 | 0.099305 | 0.484894 | SERPINF2, FLNA |
|  | GO:0044822~poly(A) RNA binding | 2 | 0.099305 | 0.646109 | HNRNPCL1, FLNA |
|  | GO:0043394~proteoglycan binding | 1 | 0.049652 | 1 | FCN2 |

**Term**: Functional class or cellular localization, **Gene**: corresponding gene and protein code to term, **BP**: biological process, **CC**: cellular component, **MF**: Molecular Function

**Table S3 Differential proteins between F2 and CON stage of hepatic fibrosis.**

|  | Term | Count | % | PValue | Genes |
| --- | --- | --- | --- | --- | --- |
| B  P | GO:0003094~glomerular filtration | 2 | 9.52381 | 9.61E-03 | IGHA1, MCAM |
|  | GO:0010977~negative regulation of neuron projection development | 2 | 9.52381 | 4.62E-02 | LRP1, CRMP1 |
|  | GO:0043524~negative regulation of neuron apoptotic process | 2 | 9.52381 | 1.32E-01 | GPI, LRP1 |
|  | GO:0007010~cytoskeleton organization | 2 | 9.52381 | 1.59E-01 | CFL1, GAN |
|  | GO:0006898~receptor-mediated endocytosis | 2 | 9.52381 | 1.82E-01 | LRP1, IGHA1 |
|  | GO:0001701~in utero embryonic development | 2 | 9.52381 | 1.83E-01 | GPI, HEG1 |
|  | GO:0001525~angiogenesis | 2 | 9.52381 | 2.14E-01 | GPI, MCAM |
|  | GO:0008283~cell proliferation | 2 | 9.52381 | 3.28E-01 | LRP1, SCRIB |
|  | GO:0006955~immune response | 2 | 9.52381 | 3.67E-01 | IL1RAP, IGHA1 |
|  | GO:0045087~innate immune response | 2 | 9.52381 | 3.73E-01 | IL1RAP, IGHA1 |
| C  C | GO:0070062~extracellular exosome | 10 | 47.61905 | 1.62E-03 | CFAP70, GPI, CILP2, SERPINA10, CFL1, IGHA1, IGFALS, UMOD, SCRIB, ORM2 |
|  | GO:0005615~extracellular space | 7 | 33.33333 | 2.53E-03 | GPI, SERPINA10, CFL1, IGHA1, IGFALS, MCAM, ORM2 |
|  | GO:0009897~external side of plasma membrane | 3 | 14.28571 | 2.25E-02 | IGHA1, HEG1, MCAM |
|  | GO:0005576~extracellular region | 6 | 28.57143 | 2.70E-02 | IL1RAP, IGHA1, IGFALS, HEG1, UMOD, ORM2 |
|  | GO:0060170~ciliary membrane | 2 | 9.52381 | 5.04E-02 | GPI, UMOD |
|  | GO:0005925~focal adhesion | 3 | 14.28571 | 6.77E-02 | LRP1, CFL1, MCAM |
|  | GO:0005929~cilium | 2 | 9.52381 | 1.53E-01 | CFAP70, UMOD |
|  | GO:0072562~blood microparticle | 2 | 9.52381 | 0.154308 | IGHA1, ORM2 |
|  | GO:0005911~cell-cell junction | 2 | 9.52381 | 0.172841 | HEG1, SCRIB |
|  | GO:0016323~basolateral plasma membrane | 2 | 9.52381 | 0.180145 | UMOD, SCRIB |
| M  F | GO:0005509~calcium ion binding | 3 | 14.28571 | 1.61E-01 | LRP1, HEG1, UMOD |
|  | GO:0098641~cadherin binding involved in cell-cell adhesion | 2 | 9.52381 | 2.55E-01 | DHX29, SCRIB |
|  | GO:0044822~poly(A) RNA binding | 2 | 9.52381 | 6.92E-01 | LRP1, DHX29 |
|  | GO:0005524~ATP binding | 2 | 9.52381 | 7.93E-01 | DHX29, SPATA5 |
|  | GO:0005515~protein binding | 8 | 38.09524 | 8.73E-01 | TGOLN2, LRP1, CRMP1, CFL1, GAN, NCAPH2, RBFA, SCRIB |
|  | GO:0031625~ubiquitin protein ligase binding | 1 | 4.761905 | 1 | GPI |
|  | GO:0004347~glucose-6-phosphate isomerase activity | 1 | 4.761905 | 1 | GPI |
|  | GO:0004908~interleukin-1 receptor activity | 1 | 4.761905 | 1 | IL1RAP |
|  | GO:0008201~heparin binding | 1 | 4.761905 | 1 | SERPINA10 |
|  | GO:0070325~lipoprotein particle receptor binding | 1 | 4.761905 | 1 | LRP1 |

**Term**: Functional class or cellular localization, **Gene**: corresponding gene and protein code to term, **BP**: biological process, **CC**: cellular component, **MF**: Molecular Function

**Table S4 Differential proteins between F1 and F2 stage of hepatic fibrosis**.

|  | Term | Count | % | PValue | Genes |
| --- | --- | --- | --- | --- | --- |
| B  P | GO:0002576~platelet degranulation | 3 | 0.094877 | 5.95E-03 | LGALS3BP, SOD1, CALM2 |
|  | GO:0001975~response to amphetamine | 2 | 0.063251 | 3.45E-02 | SOD1, CALM2 |
|  | GO:0071902~positive regulation of protein serine/  threonine kinase activity | 2 | 0.063251 | 3.89E-02 | LTF, CALM2 |
|  | GO:0001895~retina homeostasis | 2 | 0.063251 | 4.43E-02 | LTF, SOD1 |
|  | GO:0019722~calcium-mediated signaling | 2 | 0.063251 | 5.62E-02 | TREML1, CALM2 |
|  | GO:0006869~lipid transport | 2 | 0.063251 | 8.26E-02 | LPA, PLTP |
|  | GO:0030324~lung development | 2 | 0.063251 | 8.26E-02 | RBP4, HEG1 |
|  | GO:0035264~multicellular organism growth | 2 | 0.063251 | 8.68E-02 | STIL, HEG1 |
|  | GO:0045471~response to ethanol | 2 | 0.063251 | 1.12E-01 | RBP4, SOD1 |
|  | GO:0006629~lipid metabolic process | 2 | 0.063251 | 1.64E-01 | LPA, PLTP |
| C  C | GO:0005576~extracellular region | 9 | 0.28463 | 1.13E-04 | RBP4, LGALS3BP, LPA, LTF, SAA4, HEG1, SOD1, PLTP, CALM2 |
|  | GO:0070062~extracellular exosome | 11 | 0.347881 | 1.81E-04 | RBP4, LGALS3BP, KRT16, LTF, SAA4, CTDSP1, LMAN2, APMAP, SOD1, ZNHIT6, CALM2 |
|  | GO:0005615~extracellular space | 7 | 0.221379 | 1.89E-03 | RBP4, LGALS3BP, LTF, SAA4, LMAN2, SOD1, PLTP |
|  | GO:0009986~cell surface | 4 | 0.126502 | 1.78E-02 | LTF, LMAN2, APMAP, TREML1 |
|  | GO:0043234~protein complex | 3 | 0.094877 | 6.77E-02 | RBP4, LTF, SOD1 |
|  | GO:0016020~membrane | 5 | 0.158128 | 1.89E-01 | LGALS3BP, PDLIM5, NCAPH2, LMAN2, APMAP |
|  | GO:0031012~extracellular matrix | 2 | 0.063251 | 2.68E-01 | LGALS3BP, SOD1 |
|  | GO:0005813~centrosome | 2 | 0.063251 | 0.362141 | STIL, CALM2 |
|  | GO:0005829~cytosol | 5 | 0.158128 | 0.463689 | STIL, RBP4, PDLIM5, SOD1, CALM2 |
|  | GO:0005737~cytoplasm | 7 | 0.221379 | 0.47405 | STIL, PDLIM5, LTF, SOD1, LRWD1, TREML1, CALM2 |
| M  F | GO:0043539~protein serine/threonine kinase activator activity | 2 | 0.063251 | 2.01E-02 | LTF, CALM2 |
|  | GO:0008270~zinc ion binding | 4 | 0.126502 | 1.40E-01 | PDLIM5, ZNF407, SOD1, ZDBF2 |
|  | GO:0008201~heparin binding | 2 | 0.063251 | 1.66E-01 | LPA, LTF |
|  | GO:0046872~metal ion binding | 5 | 0.158128 | 1.97E-01 | ZNF407, CTDSP1, LMAN2, SOD1, ZNHIT6 |
|  | GO:0004252~serine-type endopeptidase activity | 2 | 0.063251 | 2.51E-01 | LPA, LTF |
|  | GO:0005515~protein binding | 12 | 0.379507 | 0.390836 | STIL, RBP4, LPA, KRT16, PDLIM5, LTF, CTDSP1, NCAPH2, SOD1, LRWD1, ZNHIT6, CALM2 |
|  | GO:0005509~calcium ion binding | 2 | 0.063251 | 0.561805 | HEG1, CALM2 |
|  | GO:0042802~identical protein binding | 2 | 0.063251 | 0.578006 | SOD1, ZNHIT6 |
|  | GO:0003676~nucleic acid binding | 2 | 0.063251 | 0.681117 | ZNF407, ZDBF2 |
|  | GO:0003677~DNA binding | 2 | 0.063251 | 0.862666 | ZNF407, LTF |

**Term**: Functional class or cellular localization, **Gene**: corresponding gene and protein code to term, **BP**: biological process, **CC**: cellular component, **MF**: Molecular Function

**Table S5 Differential proteins between F2 and F3 stage of hepatic fibrosis**.

|  | Term | Count | % | PValue | Genes |
| --- | --- | --- | --- | --- | --- |
| B  P | GO:0030198~extracellular matrix organization | 6 | 0.100301 | 8.59E-05 | TNR, COL3A1, SERPINE1, COL1A2, COL1A1, LAMC1 |
|  | GO:0002576~platelet degranulation | 5 | 0.083584 | 9.31E-05 | SELP, LGALS3BP, SERPINE1, CFD, ORM2 |
|  | GO:0061045~negative regulation of wound healing | 3 | 0.05015 | 2.85E-04 | APCS, SERPINE1, CD109 |
|  | GO:0007155~cell adhesion | 7 | 0.117018 | 6.12E-04 | SELP, LGALS3BP, TNR, COL1A1, LAMC1, CDH5, MYH10 |
|  | GO:0050900~leukocyte migration | 4 | 0.066867 | 2.83E-03 | SELP, PPIA, COL1A2, COL1A1 |
|  | GO:0030199~collagen fibril organization | 3 | 0.05015 | 3.69E-03 | COL3A1, COL1A2, COL1A1 |
|  | GO:0071230~cellular response to amino acid stimulus | 3 | 0.05015 | 5.32E-03 | COL3A1, COL1A2, COL1A1 |
|  | GO:0030574~collagen catabolic process | 3 | 0.05015 | 9.68E-03 | COL3A1, COL1A2, COL1A1 |
|  | GO:0048525~negative regulation of viral process | 2 | 0.033434 | 1.39E-02 | APCS, LTF |
|  | GO:0007179~transforming growth factor beta receptor signaling pathway | 3 | 0.05015 | 1.93E-02 | COL3A1, COL1A2, CDH5 |
| C  C | GO:0005615~extracellular space | 20 | 0.334336 | 4.68E-12 | SELP, C9ORF72, APCS, C4A, CPQ, COL3A1, CD109, LMAN2, FUCA2, LGALS3BP, PPIA, SERPINE1, COL1A2, LTF, CAT, COL1A1, LAMC1, CFD, CTBS, ORM2 |
|  | GO:0070062~extracellular exosome | 22 | 0.36777 | 3.07E-08 | TUFM, GNPTG, APCS, C4A, CPQ, CDHR2, UMOD, LMAN2, FUCA2, LGALS3BP, HIST1H4A, PPIA, SERPINE1, COL1A2, LTF, FABP1, CAT, LAMC1, CFD, CTBS, ORM2, MYH10 |
|  | GO:0005576~extracellular region | 17 | 0.284186 | 6.76E-08 | APCS, CNDP1, C4A, COL3A1, UMOD, LGALS3BP, HIST1H4A, PPIA, TNR, IL1RAP, SERPINE1, COL1A2, LTF, COL1A1, LAMC1, CFD, ORM2 |
|  | GO:0031012~extracellular matrix | 8 | 0.133735 | 2.74E-06 | LGALS3BP, APCS, HIST1H4A, COL3A1, SERPINE1, COL1A2, COL1A1, LAMC1 |
|  | GO:0072562~blood microparticle | 4 | 0.066867 | 4.17E-03 | LGALS3BP, APCS, C4A, ORM2 |
|  | GO:0005584~collagen type I trimer | 2 | 0.033434 | 4.28E-03 | COL1A2, COL1A1 |
|  | GO:0031093~platelet alpha granule lumen | 3 | 0.05015 | 6.17E-03 | SERPINE1, CFD, ORM2 |
|  | GO:0005764~lysosome | 4 | 0.066867 | 0.012385 | C9ORF72, CPQ, CAT, CTBS |
|  | GO:0016020~membrane | 11 | 0.183885 | 0.015145 | TUFM, ATXN2, SELP, LGALS3BP, HIST1H4A, PPIA, IL1RAP, NCAPH2, CAT, LMAN2, CDH5 |
|  | GO:0005581~collagen trimer | 3 | 0.05015 | 0.016548 | COL3A1, COL1A2, COL1A1 |
| M  F | GO:0005201~extracellular matrix structural constituent | 5 | 0.083584 | 1.51E-05 | COL3A1, COL1A2, UMOD, COL1A1, LAMC1 |
|  | GO:0048407~platelet-derived growth factor binding | 3 | 0.05015 | 2.68E-04 | COL3A1, COL1A2, COL1A1 |
|  | GO:0070573~metallodipeptidase activity | 2 | 0.033434 | 1.79E-02 | CNDP1, CPQ |
|  | GO:0001849~complement component C1q binding | 2 | 0.033434 | 2.01E-02 | APCS, C4A |
|  | GO:0046790~virion binding | 2 | 0.033434 | 2.23E-02 | APCS, PPIA |
|  | GO:0004180~carboxypeptidase activity | 2 | 0.033434 | 0.037604 | CNDP1, CPQ |
|  | GO:0016209~antioxidant activity | 2 | 0.033434 | 0.044095 | FABP1, CAT |
|  | GO:0030246~carbohydrate binding | 3 | 0.05015 | 0.071813 | SELP, APCS, LMAN2 |
|  | GO:0005509~calcium ion binding | 5 | 0.083584 | 0.076296 | APCS, CDHR2, UMOD, ZZEF1, CDH5 |
|  | GO:0004866~endopeptidase inhibitor activity | 2 | 0.033434 | 0.086295 | C4A, CD109 |

**Term**: Functional class or cellular localization, **Gene**: corresponding gene and protein code to term, **BP**: biological process, **CC**: cellular component, **MF**: Molecular Function

**Table S6 Differential proteins between F3 and F4 stage of hepatic fibrosis.**

|  | Term | Count | % | PValue | Genes |
| --- | --- | --- | --- | --- | --- |
| B  P | GO:0002576~platelet degranulation | 7 | 0.105948 | 2.69E-07 | KNG1, SERPINF2, PSAP, SERPINE1, SERPING1, CFD, AHSG |
|  | GO:0010951~negative regulation of endopeptidase activity | 5 | 0.075677 | 2.79E-04 | KNG1, SERPINF2, SERPINE1, SERPING1, AHSG |
|  | GO:0042730~fibrinolysis | 3 | 0.045406 | 1.37E-03 | SERPINF2, SERPINE1, SERPING1 |
|  | GO:0006956~complement activation | 4 | 0.060542 | 1.53E-03 | CFHR1, IGLV1-51, IGHG4, CFD |
|  | GO:0034332~adherens junction organization | 3 | 0.045406 | 4.22E-03 | CADM1, CDH2, CDH5 |
|  | GO:0007156~homophilic cell adhesion via plasma membrane adhesion molecules | 4 | 0.060542 | 8.16E-03 | CADM1, CDHR2, CDH2, CDH5 |
|  | GO:0044331~cell-cell adhesion mediated by cadherin | 2 | 0.030271 | 1.04E-02 | CDHR2, CDH2 |
|  | GO:0005975~carbohydrate metabolic process | 4 | 0.060542 | 1.06E-02 | GPI, LDHA, TALDO1, FUCA2 |
|  | GO:0010757~negative regulation of plasminogen activation | 2 | 0.030271 | 1.30E-02 | SERPINF2, SERPINE1 |
|  | GO:0051918~negative regulation of fibrinolysis | 2 | 0.030271 | 2.59E-02 | SERPINF2, SERPINE1 |
| C  C | GO:0070062~extracellular exosome | 29 | 0.438928 | 5.39E-13 | LDHA, YWHAZ, TALDO1, IGHG4, CADM1, IL6ST, JCHAIN, CDH2, TPM3, AHSG, CFHR1, SERPINE1, FGL2, CFD, KNG1, IGLV1-51, PSAP, PODXL, CDHR2, SERPING1, FUCA2, VASP, SOD3, GPI, CORO1A, CDKL1, SERPINF2, HIST1H3A, LCP1 |
|  | GO:0005615~extracellular space | 18 | 0.272438 | 5.35E-09 | KNG1, YWHAZ, IGHG4, PSAP, IL6ST, PODXL, JCHAIN, SERPING1, FUCA2, SOD3, AHSG, CFHR1, GPI, HIST1H2BK, SERPINF2, SERPINE1, CFD, LCP1 |
|  | GO:0072562~blood microparticle | 8 | 0.121084 | 7.23E-08 | CFHR1, KNG1, YWHAZ, IGHG4, SERPINF2, JCHAIN, SERPING1, AHSG |
|  | GO:0031093~platelet alpha granule lumen | 6 | 0.090813 | 2.06E-07 | KNG1, SERPINF2, SERPINE1, SERPING1, CFD, AHSG |
|  | GO:0005576~extracellular region | 13 | 0.196761 | 3.15E-04 | KNG1, IGLV1-51, IGHG4, SERPINF2, IL6ST, PSAP, HIST1H3A, SERPINE1, JCHAIN, SERPING1, CFD, SOD3, AHSG |
|  | GO:0005911~cell-cell junction | 5 | 0.075677 | 7.75E-04 | PARD3, CORO1A, CADM1, CDH2, CDH5 |
|  | GO:0005913~cell-cell adherens junction | 6 | 0.090813 | 1.04E-03 | LDHA, YWHAZ, CADM1, HIST1H3A, CDH2, VASP |
|  | GO:0030027~lamellipodium | 4 | 0.060542 | 0.006758 | CORO1A, PODXL, CDH2, VASP |
|  | GO:0005884~actin filament | 3 | 0.045406 | 0.010761 | CORO1A, LCP1, TPM3 |
|  | GO:0030175~filopodium | 3 | 0.045406 | 0.012739 | PODXL, LCP1, VASP |
| M  F | GO:0005515~protein binding | 32 | 0.484335 | 5.86E-03 | LDHA, YWHAZ, PARD3, TALDO1, CADM1, IL6ST, CDH2, CDH5, TPM3, SERPINE1, LARS, TIE1, KNG1, RPGR, PSAP, PODXL, CDHR2, SERPING1, CCDC137, FUCA2, VASP, SOD3, TRDN, ATXN2, CORO1A, SERPINF2, PSMC2, LSAMP, HIST1H3A, MGA, TBX18, ADRA1D |
|  | GO:0019903~protein phosphatase binding | 3 | 0.045406 | 1.12E-02 | PARD3, CDH2, CDH5 |
|  | GO:0005102~receptor binding | 5 | 0.075677 | 1.22E-02 | TRDN, KNG1, CADM1, SERPINE1, CDH5 |
|  | GO:0048029~monosaccharide binding | 2 | 0.030271 | 1.77E-02 | GPI, TALDO1 |
|  | GO:0004867~serine-type endopeptidase inhibitor activity | 3 | 0.045406 | 2.53E-02 | SERPINF2, SERPINE1, SERPING1 |
|  | GO:0003823~antigen binding | 3 | 0.045406 | 0.028304 | IGLV1-51, IGHG4, JCHAIN |
|  | GO:0042803~protein homodimerization activity | 6 | 0.090813 | 0.03701 | CORO1A, CADM1, SERPINF2, IL6ST, JCHAIN, TBX18 |
|  | GO:0098641~cadherin binding involved in cell-cell adhesion | 4 | 0.060542 | 0.037382 | LDHA, YWHAZ, HIST1H3A, VASP |
|  | GO:0051015~actin filament binding | 3 | 0.045406 | 0.044486 | CORO1A, LCP1, TPM3 |
|  | GO:0034987~immunoglobulin receptor binding | 2 | 0.030271 | 0.064208 | IGHG4, JCHAIN |

**Term**: Functional class or cellular localization, **Gene**: corresponding gene and protein code to term, **BP**: biological process, **CC**: cellular component, **MF**: Molecular Function

**Table S7KEGG pathway analysis of the identified differential proteins of hepatic fibrosis**

|  | Pathway | Genes |
| --- | --- | --- |
| Between F1 and F2 | hsa04979 Cholesterol metabolism - Homo sapiens (human) (2) | hsa:4018 LPA; lipoprotein(a) hsa:5360 PLTP; phospholipid transfer protein |
| Between F2 and F3 | hsa05146 Amoebiasis - Homo sapiens (human) (4) | hsa:1277 COL1A1; collagen type I alpha 1 chain hsa:1278 COL1A2; collagen type I alpha 2 chain hsa:1281 COL3A1; collagen type III alpha 1 chain hsa:3915 LAMC1; laminin subunit gamma 1 |
|  | hsa04510 Focal adhesion - Homo sapiens (human) (4) | hsa:1277 COL1A1; collagen type I alpha 1 chain hsa:1278 COL1A2; collagen type I alpha 2 chain hsa:3915 LAMC1; laminin subunit gamma 1 hsa:7143 TNR; tenascin R |
|  | hsa04151 PI3K-Akt signaling pathway - Homo sapiens (human) (4) | hsa:1277 COL1A1; collagen type I alpha 1 chain hsa:1278 COL1A2; collagen type I alpha 2 chain hsa:3915 LAMC1; laminin subunit gamma 1 hsa:7143 TNR; tenascin R |
|  | hsa05165 Human papillomavirus infection - Homo sapiens (human) (4) | hsa:1277 COL1A1; collagen type I alpha 1 chain hsa:1278 COL1A2; collagen type I alpha 2 chain hsa:3915 LAMC1; laminin subunit gamma 1 hsa:7143 TNR; tenascin R |
|  | hsa04933 AGE-RAGE signaling pathway in diabetic complications - Homo sapiens (human) (4) | hsa:1277 COL1A1; collagen type I alpha 1 chain hsa:1278 COL1A2; collagen type I alpha 2 chain hsa:1281 COL3A1; collagen type III alpha 1 chain hsa:5054 SERPINE1; serpin family E member 1 |
|  | hsa04512 ECM-receptor interaction - Homo sapiens (human) (4) | hsa:1277 COL1A1; collagen type I alpha 1 chain hsa:1278 COL1A2; collagen type I alpha 2 chain hsa:3915 LAMC1; laminin subunit gamma 1 hsa:7143 TNR; tenascin R |
|  | hsa04926 Relaxin signaling pathway - Homo sapiens (human) (3) | hsa:1277 COL1A1; collagen type I alpha 1 chain hsa:1278 COL1A2; collagen type I alpha 2 chain hsa:1281 COL3A1; collagen type III alpha 1 chain |
|  | hsa05150 Staphylococcus aureus infection - Homo sapiens (human) (3) | hsa:720 C4A; complement C4A (Rodgers blood group) hsa:1675 CFD; complement factor D hsa:6403 SELP; selectin P |
|  | hsa04611 Platelet activation - Homo sapiens (human) (3) | hsa:1277 COL1A1; collagen type I alpha 1 chain hsa:1278 COL1A2; collagen type I alpha 2 chain hsa:1281 COL3A1; collagen type III alpha 1 chain |
|  | hsa04610 Complement and coagulation cascades - Homo sapiens (human) (3) | hsa:720 C4A; complement C4A (Rodgers blood group) hsa:1675 CFD; complement factor D hsa:5054 SERPINE1; serpin family E member 1 |
|  | hsa04974 Protein digestion and absorption - Homo sapiens (human) (3) | hsa:1277 COL1A1; collagen type I alpha 1 chain hsa:1278 COL1A2; collagen type I alpha 2 chain hsa:1281 COL3A1; collagen type III alpha 1 chain |
|  | hsa05206 MicroRNAs in cancer - Homo sapiens (human) (2) | hsa:9493 KIF23; kinesin family member 23 hsa:7143 TNR; tenascin R |
|  | hsa04514 Cell adhesion molecules (CAMs) - Homo sapiens (human) (2) | hsa:1003 CDH5; cadherin 5 hsa:6403 SELP; selectin P |
|  | hsa05322 Systemic lupus erythematosus - Homo sapiens (human) (2) | hsa:720 C4A; complement C4A (Rodgers blood group) hsa:8359 HIST1H4A; histone cluster 1 H4 family member a |
| Between F3 and F4 | hsa04610 Complement and coagulation cascades - Homo sapiens (human) (5) | hsa:1675 CFD; complement factor D hsa:3827 KNG1; kininogen 1 hsa:5054 SERPINE1; serpin family E member 1 hsa:5345 SERPINF2; serpin family F member 2 hsa:710 SERPING1; serpin family G member 1 |
|  | hsa01100 Metabolic pathways - Homo sapiens (human) (3) | hsa:2821 GPI; glucose-6-phosphate isomerase hsa:3939 LDHA; lactate dehydrogenase A hsa:6888 TALDO1; transaldolase 1 |
|  | hsa05203 Viral carcinogenesis - Homo sapiens (human) (3) | hsa:85236 HIST1H2BK; histone cluster 1 H2B family member k hsa:3572 IL6ST; interleukin 6 signal transducer hsa:7534 YWHAZ; tyrosine 3-monooxygenase/tryptophan 5-monooxygenase activation protein zeta |
|  | hsa04514 Cell adhesion molecules (CAMs) - Homo sapiens (human) (3) | hsa:23705 CADM1; cell adhesion molecule 1 hsa:1000 CDH2; cadherin 2 hsa:1003 CDH5; cadherin 5 |
|  | hsa04390 Hippo signaling pathway - Homo sapiens (human) (3) | hsa:56288 PARD3; par-3 family cell polarity regulator hsa:5054 SERPINE1; serpin family E member 1 hsa:7534 YWHAZ; tyrosine 3-monooxygenase/tryptophan 5-monooxygenase activation protein zeta |
|  | hsa04261 Adrenergic signaling in cardiomyocytes - Homo sapiens (human) (2) | hsa:146 ADRA1D; adrenoceptor alpha 1D hsa:7170 TPM3; tropomyosin 3 |
|  | hsa00030 Pentose phosphate pathway - Homo sapiens (human) (2) | hsa:2821 GPI; glucose-6-phosphate isomerase hsa:6888 TALDO1; transaldolase 1 |
|  | hsa05169 Epstein-Barr virus infection - Homo sapiens (human) (2) | hsa:5701 PSMC2; proteasome 26S subunit, ATPase 2 hsa:7534 YWHAZ; tyrosine 3-monooxygenase/tryptophan 5-monooxygenase activation protein zeta |
|  | hsa05034 Alcoholism - Homo sapiens (human) (2) | hsa:85236 HIST1H2BK; histone cluster 1 H2B family member k hsa:8350 HIST1H3A; histone cluster 1 H3 family member a |
|  | hsa05200 Pathways in cancer - Homo sapiens (human) (2) | hsa:3572 IL6ST; interleukin 6 signal transducer hsa:7170 TPM3; tropomyosin 3 |
|  | hsa04080 Neuroactive ligand-receptor interaction - Homo sapiens (human) (2) | hsa:146 ADRA1D; adrenoceptor alpha 1D hsa:56288 PARD3; par-3 family cell polarity regulator |
|  | hsa04015 Rap1 signaling pathway - Homo sapiens (human) (2) | hsa:56288 PARD3; par-3 family cell polarity regulator hsa:7408 VASP; vasodilator stimulated phosphoprotein |
|  | hsa04066 HIF-1 signaling pathway - Homo sapiens (human) (2) | hsa:3939 LDHA; lactate dehydrogenase A hsa:5054 SERPINE1; serpin family E member 1 |
|  | hsa01200 Carbon metabolism - Homo sapiens (human) (2) | hsa:2821 GPI; glucose-6-phosphate isomerase hsa:6888 TALDO1; transaldolase 1 |
|  | hsa04022 cGMP-PKG signaling pathway - Homo sapiens (human) (2) | hsa:146 ADRA1D; adrenoceptor alpha 1D hsa:7408 VASP; vasodilator stimulated phosphoprotein |
|  | hsa00010 Glycolysis / Gluconeogenesis - Homo sapiens (human) (2) | hsa:2821 GPI; glucose-6-phosphate isomerase hsa:3939 LDHA; lactate dehydrogenase A |
|  | hsa04530 Tight junction - Homo sapiens (human) (2) | hsa:56288 PARD3; par-3 family cell polarity regulator hsa:7408 VASP; vasodilator stimulated phosphoprotein |
|  | hsa05322 Systemic lupus erythematosus - Homo sapiens (human) (2) | hsa:85236 HIST1H2BK; histone cluster 1 H2B family member k hsa:8350 HIST1H3A; histone cluster 1 H3 family member a |
|  | hsa04670 Leukocyte transendothelial migration - Homo sapiens (human) (2) | hsa:1003 CDH5; cadherin 5 hsa:7408 VASP; vasodilator stimulated phosphoprotein |

**Pathway**: Signaling pathway that change between different stage**, Genes:** corresponding gene and protein code to pathway

**Table S8 PPI network analysis between hepatic fibrosis stages**

|  | GENE | Degree |
| --- | --- | --- |
| Between F1 and control | SERPINF2 | 1 |
|  | TRPV4 | 1 |
|  | RBP4 | 1 |
|  | SAA4 | 1 |
| Between F2 and control | CRMP1 | 2 |
|  | RBFA | 1 |
|  | SCRIB | 1 |
|  | DHX29 | 2 |
|  | IGFALS | 1 |
|  | CFL1 | 1 |
| Between F1 and F2 | CALM2 | 5 |
|  | SOD1 | 3 |
|  | PDLIM5 | 3 |
|  | LRWD1 | 2 |
|  | LRRC63 | 2 |
|  | LPA | 3 |
|  | SDPR | 2 |
|  | SAA4 | 3 |
|  | HEG1 | 1 |
|  | PLTP | 2 |
|  | RBP4 | 4 |
|  | ZNF407 | 2 |
| Between F2 and F3 | COL1A2 | 5 |
|  | COL1A1 | 7 |
|  | COL3A1 | 7 |
|  | ORM2 | 4 |
|  | SERPINE1 | 10 |
|  | LGALS3BP | 5 |
|  | CFD | 8 |
|  | APCS | 6 |
|  | LTF | 3 |
|  | CD109 | 2 |
|  | SELP | 5 |
|  | C9orf72 | 1 |
|  | ATXN2 | 2 |
|  | TNR | 3 |
|  | LAMC1 | 5 |
|  | CDH5 | 3 |
|  | UMOD | 1 |
|  | FABP1 | 3 |
|  | GNPTG | 1 |
|  | FUCA2 | 2 |
|  | C4A | 6 |
|  | CAT | 11 |
|  | CDC45 | 3 |
|  | HIST1H4A | 9 |
|  | PPIA | 12 |
|  | LRWD1 | 8 |
| Between F3 and F4 | GPI | 5 |
|  | TALDO1 | 3 |
|  | SERPING1 | 7 |
|  | KNG1 | 12 |
|  | CFD | 7 |
|  | SERPINF2 | 9 |
|  | SERPINE1 | 11 |
|  | AHSG | 6 |
|  | CDH5 | 9 |
|  | CDH2 | 10 |
|  | ADRA1D | 3 |
|  | PARD3 | 3 |
|  | HIST1H3A | 4 |
|  | HIST1H2BK | 2 |
|  | LCP1 | 6 |
|  | CORO1A | 2 |
|  | YWHAZ | 9 |
|  | FGL2 | 3 |
|  | TPM3 | 5 |
|  | LDHA | 7 |
|  | ATXN2 | 2 |
|  | VASP | 6 |
|  | TIE1 | 12 |
|  | PSMC2 | 2 |
|  | LARS | 5 |
|  | CFHR1 | 3 |
|  | FUCA2 | 1 |
|  | PODXL | 2 |
|  | TRDN | 2 |
|  | SOD3 | 4 |
|  | CCDC137 | 1 |
|  | CADM1 | 4 |
|  | IGJ | 3 |
|  | TBX18 | 1 |
|  | ZNF407 | 3 |
|  | RPGR | 2 |

**Gene**: gene code, **Degree**: the degree of differentially expressed proteins between different stage.

**Figure S1** Functional annotation of proteins with GO between F2 and F3 stage of hepatic fibrosis. Pink, green, and blue bars represent proteins functionally annotated for biological processes, cellular components, and molecular functions, respectively.


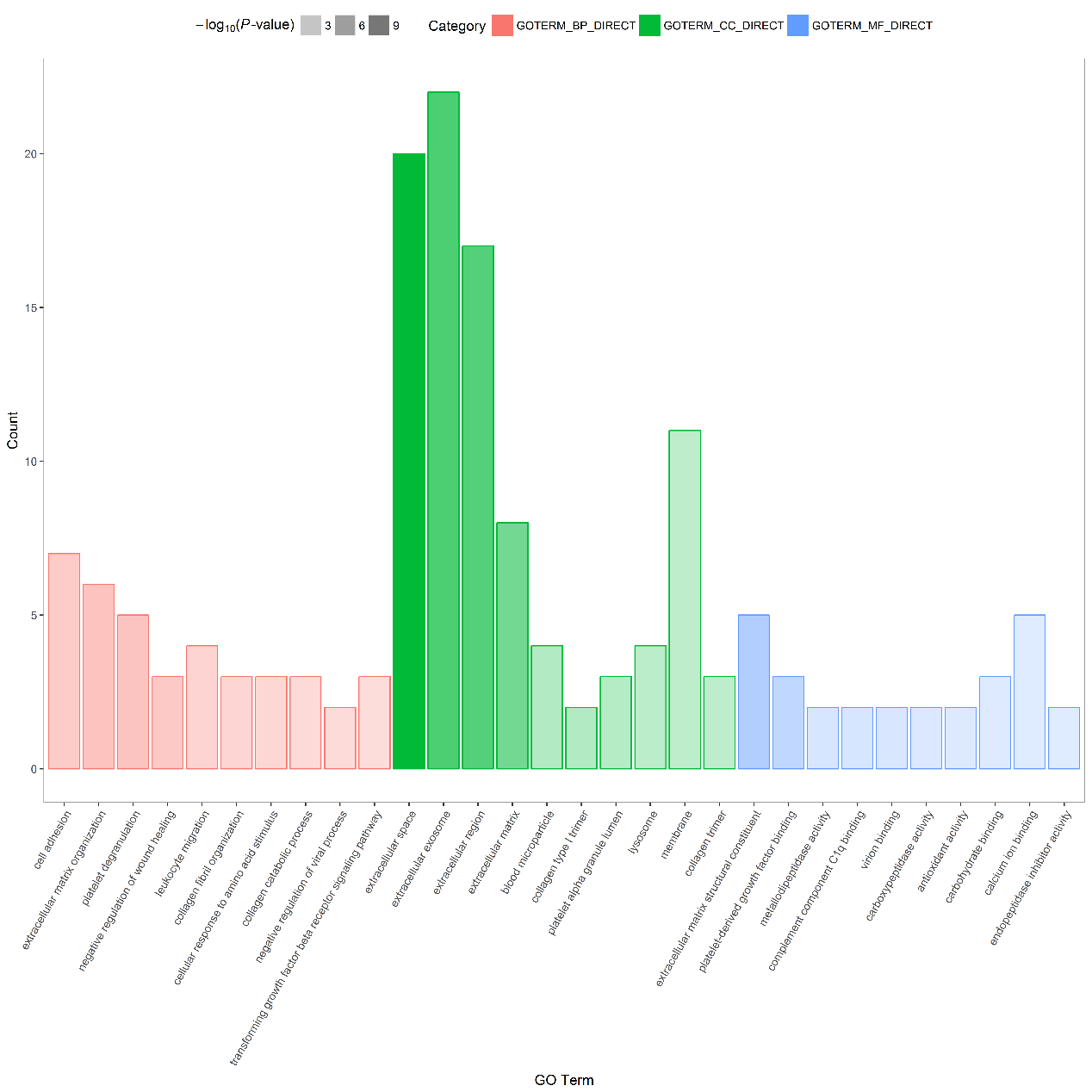


Figure S2Functional annotation of proteins with GO between F3 and F4 stage of hepatic fibrosis. Pink, green, and blue bars represent proteins functionally annotated for biological processes, cellular components, and molecular functions, respectively.


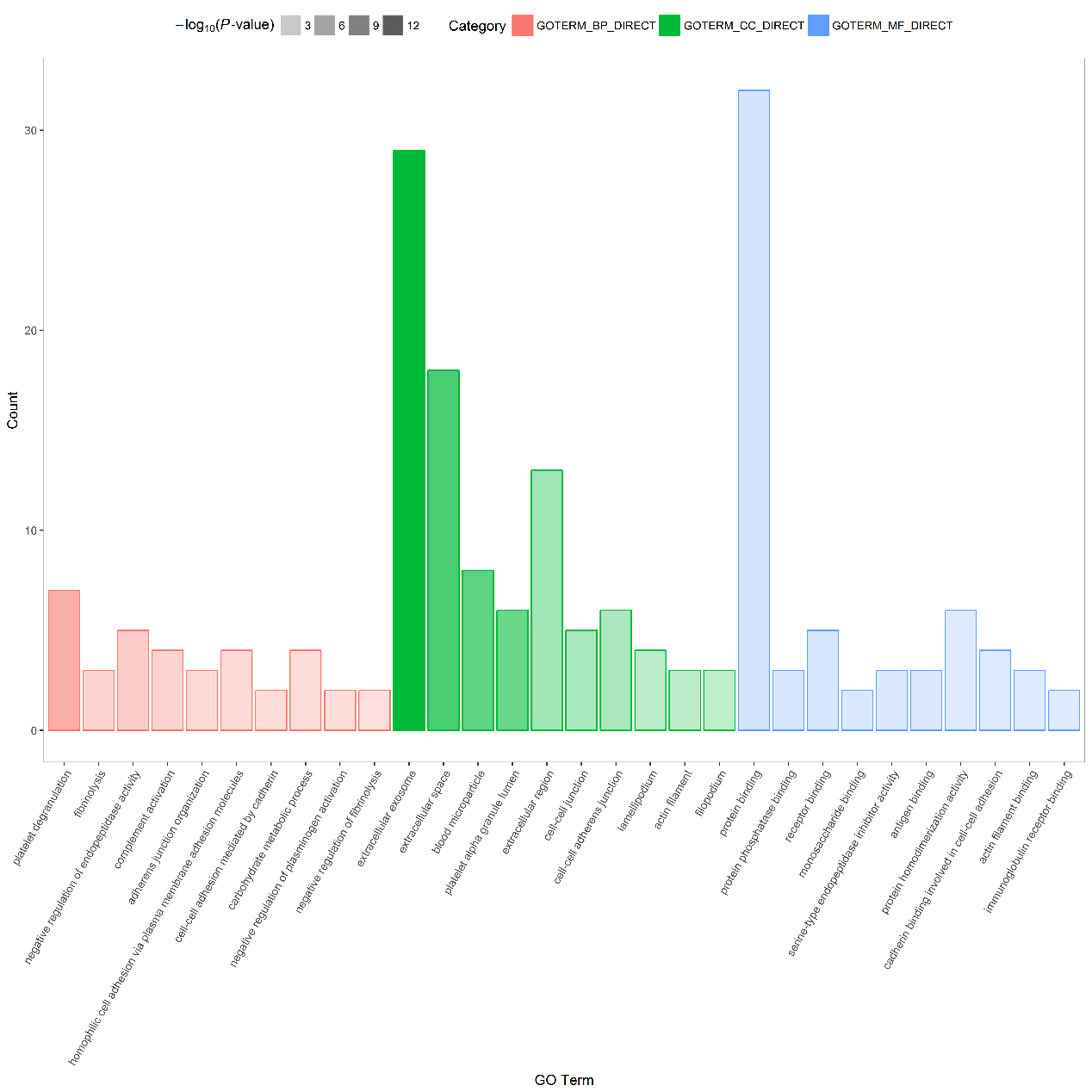


Figure S3 PPI of differentially expressed proteins between F2 and F3 stage of hepatic fibrosis.

Colors indicate the abundance intensity, with higher abundance intensity represented by a gradual increase from blue to red. The size of the circles represents the degree of differentially expressed proteins, with larger circles indicating higher degrees.


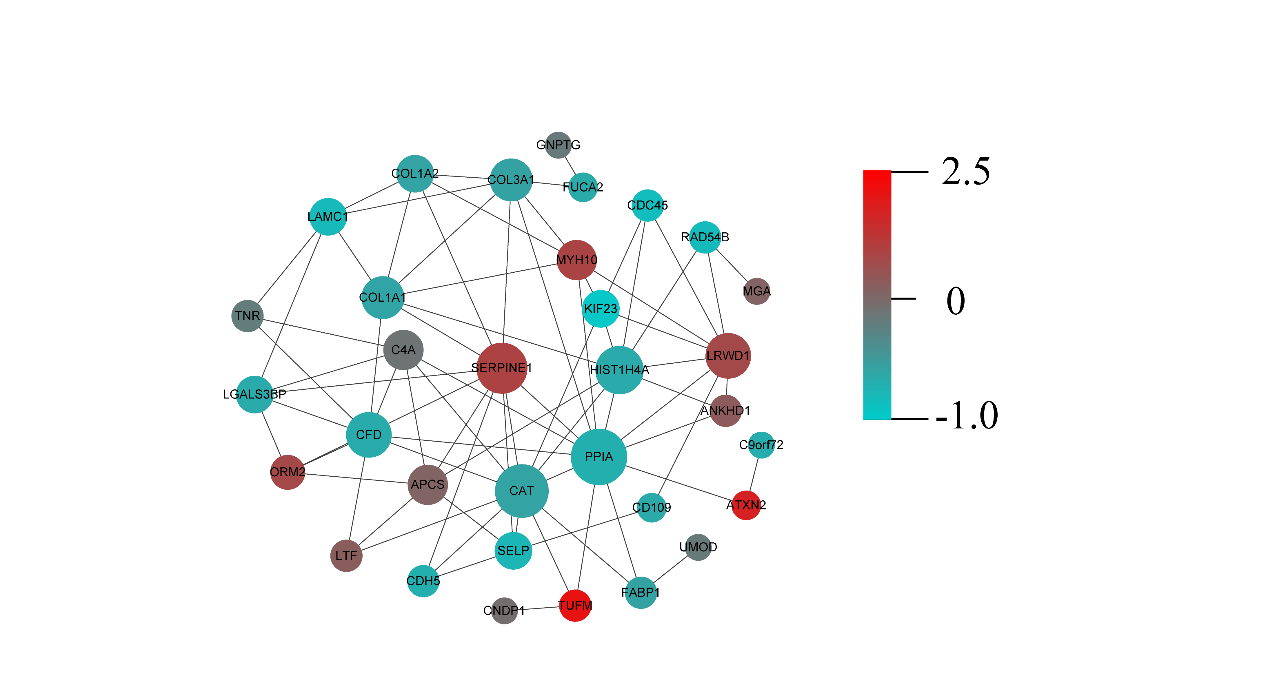


Figure S4 PPI of differentially expressed proteins between F3 and F4 stage of hepatic fibrosis.

Colors indicate the abundance intensity, with higher abundance intensity represented by a gradual increase from blue to red. The size of the circles represents the degree of differentially expressed proteins, with larger circles indicating higher degrees.


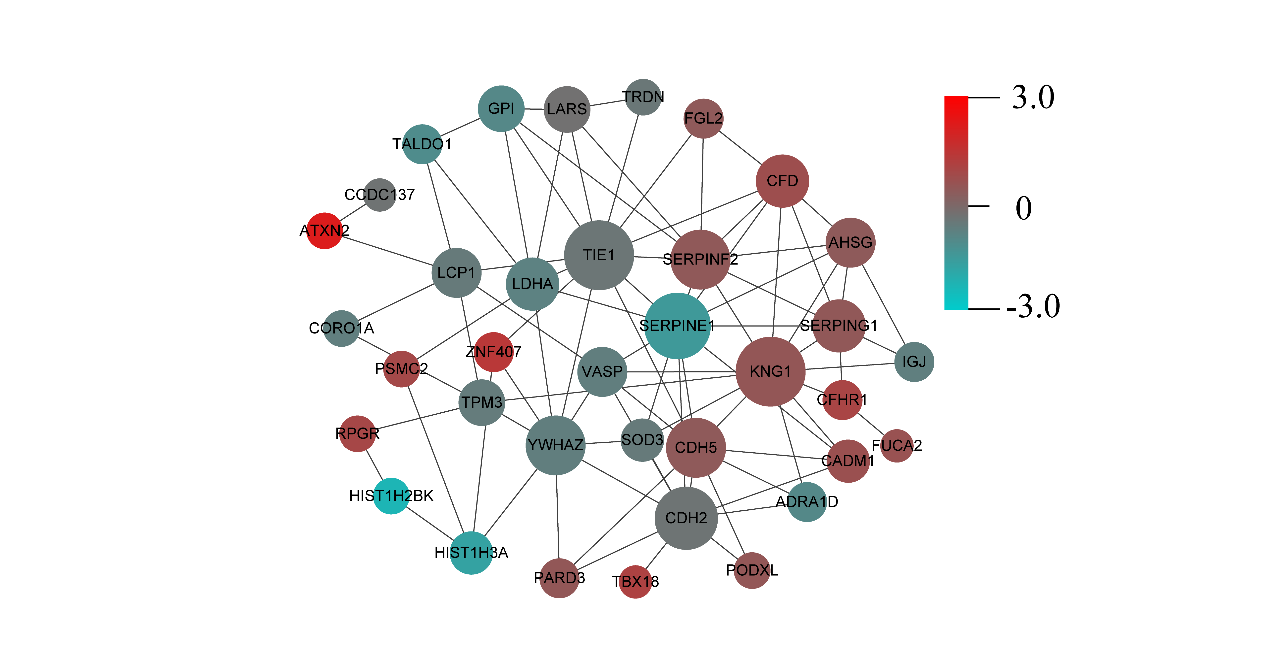

Supplement: Supplementary Materials — Table S1: the descriptions of the differential proteome. Table S2: differential proteins between F1 and CON stage of hepatic fibrosis. Table S3: differential proteins between F2 and CON stage of hepatic fibrosis. Table S4: differential proteins between F1 and F2 stage of hepatic fibrosis. Table S5: differential proteins between F2 and F3 stage of hepatic fibrosis. Table S6: differential proteins between F3 and F4 stage of hepatic fibrosis. Table S7: KEGG pathway analysis of the identified differential proteins of hepatic fibrosis. Table S8: PPI network analysis between hepatic fibrosis stages. Figure S1: functional annotation of proteins with GO between F2 and F3 stage of hepatic fibrosis. Figure S2: functional annotation of proteins with GO between F3 and F4 stage of hepatic fibrosis. Figure S3: PPI of differentially expressed proteins between F2 and F3 stage of hepatic fibrosis. Figure S4: PPI of differentially expressed proteins between F3 and F4 stage of hepatic fibrosis. [file 3580090.f1.docx]
